# Costs and Cost-Effectiveness of Malaria Control Interventions: A Systematic Literature Review

**DOI:** 10.1016/j.jval.2021.01.013

**Published:** 2021-08

**Authors:** Lesong Conteh, Kathryn Shuford, Efundem Agboraw, Mara Kont, Jan Kolaczinski, Edith Patouillard

**Affiliations:** 1 Department of Health Policy, London School of Economics and Political Science, London, England, UK; 2 School of Public Health, Imperial College London, St Mary's Campus, Paddington, England, UK; 3 Vector Biology, Liverpool School of Tropical Medicine, Liverpool, England, UK; 4 Department of Infectious Disease Epidemiology, MRC Centre for Global Infectious Disease Analysis, Imperial College London, England, UK; 5 Department of the Global Malaria Programme, World Health Organization, Geneva, Switzerland; 6 Department of Health Systems Governance and Financing, World Health Organization, Geneva, Switzerland

**Keywords:** cost-effectiveness, disease control interventions, malaria, unit cost

## Abstract

**Objectives:**

To systematically review the literature on the unit cost and cost-effectiveness of malaria control.

**Methods:**

Ten databases and gray literature sources were searched to identify evidence relevant to the period 2005 to 2018. Studies with primary financial or economic cost data from malaria endemic countries that took a provider, provider and household, or societal perspective were included.

**Results:**

We identified 103 costing studies. The majority of studies focused on individual rather than combined interventions, notably insecticide-treated bed nets and treatment, and commonly took a provider perspective. A third of all studies took place in 3 countries. The median provider economic cost of protecting 1 person per year ranged from $1.18 to $5.70 with vector control and from $0.53 to $5.97 with chemoprevention. The median provider economic cost per case diagnosed with rapid diagnostic tests was $6.06 and per case treated $9.31 or $89.93 depending on clinical severity. Other interventions did not share enough similarities to be summarized. Cost drivers were rarely reported. Cost-effectiveness of malaria control was reiterated, but care in methodological and reporting standards is required to enhance data transferability.

**Conclusions:**

Important information that can support resource allocation was reviewed. Given the variability in methods and reporting, global efforts to follow existing standards are required for the evidence to be most useful outside their study context, supplemented by guidance on options for transferring existing data across settings.

## Introduction

No significant reduction in malaria burden has been recorded since 2015, and in some countries, the disease burden is on the rise.^1^ In 2018, 6 countries accounted for more than half of all malaria cases (Nigeria, the Democratic Republic of the Congo, Uganda, Côte d’Ivoire, Mozambique, and Niger), and children under 5 years of age represented two thirds of the 405 000 malaria-related deaths globally.^1^ The level of global investments in malaria is reported to be below the estimated resource needs to achieve progress targets.^2^, ^3^, ^4^, ^5^ Under tight funding constraints, evidence on the unit cost and cost-effectiveness of malaria control interventions becomes ever more important, and how resources are allocated comes under increasing scrutiny. We update previous malaria control unit cost and cost-effectiveness reviews^6^, ^7^, ^8^, ^9^, ^10^ and widen the scope of evidence by adding new data and interventions with the aim to inform decision-making processes for national malaria control strategies.

## Methods

### Search Strategy and Selection Criteria

We searched peer-reviewed studies from Medline, Embase, Econlit, the National Health Service Economic Evaluation Database, the Cost-Effectiveness Analysis Registry, Cochrane Library, Web of Science, and the Latin American and Caribbean Health Sciences Literature and gray literature from GreyNet/OpenSIGLE, the Social Science Research Network, and the websites of the World Bank Group, the World Health Organization (WHO), the United States Agency for International Development, and Population Services International. Searches were restricted to studies published between January 1, 2005, and August 31, 2018. Before 2005, the set of malaria control interventions implemented by countries was relatively limited; some of these interventions are not recommended anymore, while others had very low coverage and have since been replaced by different commodities. Our project started in June 2018, and we stopped searches in September 2018. We used English search terms (see Appendix 1 in Supplemental Materials found at https://doi.org/10.1016/j.jval.2021.01.013) only but considered studies published in English, French, or Spanish. Reference lists of eligible studies were reviewed and topic experts consulted to identify additional articles for inclusion. Studies included had to contain primary cost data on 1 or more WHO-recommended malaria control interventions and take a provider, provider and household, or societal perspective. Excluded were studies that: (1) exclusively relied on mathematical modeling of cost data published by other studies; (2) took a household perspective only; (3) were found in poster presentation or conference abstract formats only; or (4) pertained to the health of short-term travelers from non-endemic to endemic countries. The study protocol was registered under Prospero, number CRD42018105625.

### Data Management and Analysis

Titles and abstracts were imported into the Covidence systematic review online management tool. Three reviewers independently screened titles and abstracts, retrieved full texts of potentially relevant studies, and assessed study eligibility for inclusion; discrepancies were resolved by a fourth reviewer. Review team members extracted the data independently using a table developed following discussions with investigators from the Global Health Costing Consortium.^11^ Extracted data included: the characteristics of each eligible study (first author, publication year, country name, rural/urban or mixed study setting); details of the studied intervention (type, delivery strategy, and/or platform; population targeted; number of commodities distributed or area covered if applicable); the analytical methods (study perspective, including provider/household and provider or societal; financial or economic cost; unit cost output measure, cost-effectiveness health outcome measure where applicable); and the results (unit cost or cost-effectiveness estimate; breakdown of unit cost data by cost category, by resource input, and/or activity where available).

When a study provided data for several years, only data from the most recent year were extracted. If unit cost data were not explicitly reported by studies, data on total cost and number of commodities delivered or individuals covered by an intervention were used. When the cost per treatment course was not reported by a study, we estimated it using cost per dose data reported by the study and WHO treatment recommendations to allow output cost comparability across studies. Malaria treatment at outpatient departments was considered uncomplicated malaria treatment, whereas health facility admissions were assumed to be severe cases. For graphical display, percentage unit cost category data were converted to absolute terms.

Summary statistics were calculated by intervention, perspective, and unit cost output and/or cost-effectiveness health outcome measure when more than 3 data points were available. We present economic rather than financial data to better reflect resource use. All cost data were converted to constant USD 2018.^12^ Cost data in currencies other than US dollars were first converted from the local currency to US dollars using the exchange rate at the year of costing before being inflated to 2018 USD.^13^

## Results

The search yielded 16 985 records. Using Covidence, 6505 duplicates were removed. A further 9621 records were excluded by title or abstract; 859 full-text articles were read and 754 were excluded, of which 180 were duplicates previously unidentified by Covidence, and 576 studies did not meet our inclusion criteria. A total of 103 eligible studies were identified. This section summarizes key results across eligible studies before describing in more detail results by intervention type.

### Overview of Results

**Figure 1:**
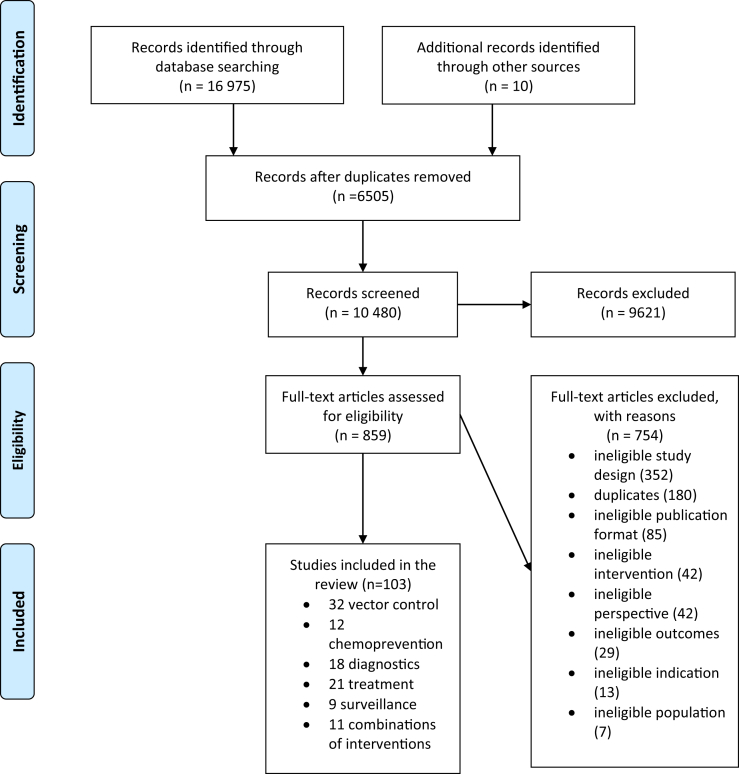
PRISMA flow chart.

**Figure 2:**
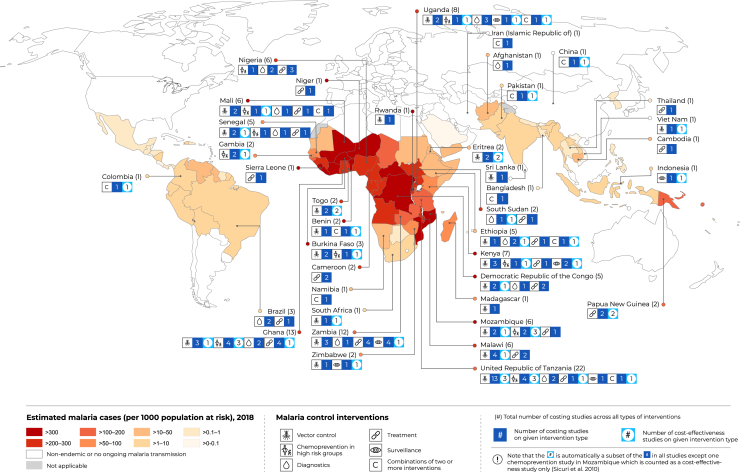
Geographical distribution of eligible studies by intervention and study type.

Eligible studies concerned vector control interventions (n = 32, 31%), chemoprevention in special risk groups (n = 12, 12%), diagnostics (n = 18, 17%), treatment (n = 21, 20%), surveillance (n = 9, 9%), and combinations of 2 or more interventions (n = 11, 11%) (Fig. 1). The number of eligible studies peaked at 14 in 2014 and 2017 (see Appendix 2 in Supplemental Materials found at https://doi.org/10.1016/j.jval.2021.01.013). The eligible studies covered a total of 39 countries, with one third of the studies concerned with the unit cost and cost-effectiveness of malaria control in 3 sub-Saharan African countries only, including Tanzania (n = 22), Ghana (n = 13), and Zambia (n = 12) (Fig. 2). Fewer studies concerned other regions, including the Eastern Mediterranean region (n = 3), the Southeast Asia region (n = 3), the Western Pacific region (n = 5), and the region of the Americas (n = 4). Less than one fifth (18%) of the eligible studies took place in 1 of the 6 countries that together accounted for more than half of all malaria cases worldwide in 2018.^1^ When interpreting the geographical distribution and intervention type of studies, however, it is important to note that among the eligible studies were both multi-country (11%) and multi-intervention (11%) studies.

From a provider perspective, the median economic cost of protecting 1 person from malaria ranged from $1.18 to $5.70 with vector control and from $0.53 to $5.97 with chemoprevention. The median provider economic cost per case diagnosed was $6.06 with rapid diagnostic test (RDT) and $2.53 with microscopy, while it was per case treated $9.31 and $89.93 for uncomplicated and severe malaria, respectively. For surveillance and combinations of interventions, the types of activities and the range of unit cost output measures used in the eligible studies did not share enough similarities to be summarized.

### Vector Control

Vector control interventions include measures against malaria-transmitting mosquitoes intended to limit mosquitoes’ ability to transmit the disease. Vector control eligible studies investigated the cost of the 2 core vector control interventions, including insecticide-treated nets (ITNs) (n = 26, 81%),^14^, ^15^, ^16^, ^17^, ^18^, ^19^, ^20^, ^21^, ^22^, ^23^, ^24^, ^25^, ^26^, ^27^, ^28^, ^29^, ^30^, ^31^, ^32^, ^33^, ^34^, ^35^, ^36^, ^37^, ^38^, ^39^ and/or indoor residual spraying (IRS) (n = 3, 9%)^23^^,^^37^^,^^40^ or larval source management (LSM) (n = 5, 16%),^41^, ^42^, ^43^, ^44^, ^45^ a supplementary vector intervention. Seven ITN,^14^^,^^19^, ^20^, ^21^^,^^29^^,^^37^, ^38^, ^39^ 1 IRS,^37^ and 1 LSM^43^ studies were also cost-effectiveness studies.

#### Insecticide-treated bed nets (ITN)

ITN are either conventionally treated nets that rely on periodic retreatment of nets by dipping into an insecticide formulation or factory-treated, pyrethroid-only, long-lasting insecticide nets (LLINs) made of netting material with insecticide incorporated within or bound around the fibers. A net needs to retain its effective biological activity for 3 years of recommended use under field conditions to qualify as an LLIN. Most ITN studies considered pyrethroid-only long-lasting insecticide nets (LLIN),^15^^,^^16^^,^^18^^,^^19^^,^^22^^,^^24^, ^25^, ^26^, ^27^, ^28^, ^29^^,^^31^, ^32^, ^33^, ^34^, ^35^, ^36^^,^^38^ the most common type of ITN currently deployed, whereas others, published before 2009, examined ITN with pyrethroid insecticide retreatment.^17^^,^^21^^,^^30^^,^^37^, ^38^, ^39^ More than half of ITN studies investigated continuous distribution, while others (n = 9, 35%) analyzed campaigns (see Appendix 3 in Supplemental Materials found at https://doi.org/10.1016/j.jval.2021.01.013).

#### Indoor-residual spraying (IRS)

IRS involves spraying interior surfaces of dwellings with a residual insecticide to kill or repel endophilic mosquitoes. Of the 3 eligible IRS studies, 1 concerned 2017 cost estimates of the United States President’s Malaria Initiative IRS programs across 12 countries^40^ while the other 2 analyzed initiatives from 2006^23^ or before 2000^37^ (see Appendix 3 in Supplemental Materials found at https://doi.org/10.1016/j.jval.2021.01.013).

#### Larval source management (LSM)

LSM involves the management of aquatic habitats, which are potential habitats for mosquito larvae, to prevent completion of development of the immature stages. LSM studies concerned larviciding,^41^, ^42^, ^43^, ^44^, ^45^ which is the regular application of biological or chemical insecticides to water bodies. All but one^42^ were published after 2010 ^41^^,^^43^, ^44^, ^45^ (see Appendix 3 in Supplemental Materials found at https://doi.org/10.1016/j.jval.2021.01.013).

#### Unit cost and cost-effectiveness of vector control interventions

From a provider perspective, the median economic cost per person protected per year (PPPY) with ITN was US$1.39 (interquartile range [IQR] 0.72),^25^, ^26^, ^27^^,^^34^, ^35^, ^36^ with IRS $5.70 (IQR 2.0)^37^^,^^39^^,^^40^ and with larviciding $1.18 (IQR 0.54) (see Appendix 3 in Supplemental Materials found at https://doi.org/10.1016/j.jval.2021.01.013).^43^, ^44^, ^45^ Additional unit economic cost measures for ITN included $1.77 (IQR 1.46) per treated net year (TNY)^18^^,^^26^^,^^27^^,^^30^^,^^34^, ^35^, ^36^ and $5.13 (IQR 3.76) per net distributed (Appendix 3).^14^^,^^16^^,^^18^^,^^20^^,^^21^^,^^25^, ^26^, ^27^^,^^29^, ^30^, ^31^, ^32^, ^33^, ^34^, ^35^, ^36^, ^37^^,^^39^ Cost category data suggest that nets represent nearly half of the cost per ITN distributed, while once excluded, personnel is the main cost category, followed by education/communication activities (IEC) and transport (see Appendix 4 in Supplemental Materials found at https://doi.org/10.1016/j.jval.2021.01.013). IRS and larviciding studies suggested insecticide to be the largest cost category, followed for IRS by project management and spray operations (see Appendix 5 in Supplemental Materials found at https://doi.org/10.1016/j.jval.2021.01.013) and for larviciding by personnel (see Appendix 6 in Supplemental Materials found at https://doi.org/10.1016/j.jval.2021.01.013).

Of all the interventions, ITNs had the most cost-effectiveness data. ITN cost-effectiveness compared with no ITN was $5.85 per episode averted (IQR 5.96)^20^ and, on average, $1281.97 (IQR 998.24) per death averted^14^^,^^20^^,^^21^^,^^37^, ^38^, ^39^ and $44.51 (IQR 35.04) per DALY averted^14^^,^^20^^,^^38^^,^^39^ from a provider perspective across several sub-Saharan African settings (Table 1). Using different insecticides in South Africa and Mozambique, IRS cost-effectiveness compared to no IRS, from the provider perspective, was $840.44 per death averted and $25.16 per DALY averted.^37^ In a high-transmission setting of Tanzania, larviciding cost-effectiveness compared to no larviciding was $2.62 per case averted, $2412.17 per death averted, and $46.87 per DALY averted from a societal perspective^43^ (see Appendix 3 in Supplemental Materials found at https://doi.org/10.1016/j.jval.2021.01.013).

**Table 1:** Summary of cost-effectiveness data from eligible studies, by health outcome measure and intervention type (constant USD 2018).

	ITN	IRS	Larviciding	IPTi	IPTp	SMC	Treatment ±	Surveillance– systems for epidemics	Surveillance–active case detection
***Provider economic cost per episode averted***									
Median/min-max/point estimate	$5.85	-	-	4.38	-	121.50	0.30	1.28-389.82	79.25
Interquartile range (IQR)	5.96	-	-	5.67	-	121.81	1.47	N/A	N/A
Number of point estimates	3	0	0	15	0	10	6	2	1
Number of studies	2^20^^,^^21^	0	0	2^55^^,^^56^	0	2^48^^,^^49^	1^92^	1^103^	1^100^
***Societal economic cost per episode averted***									
Median/min-max/point estimate	$137.34∗	-	2.62	-	-	177.34	30.99†	-	-
Interquartile range (IQR)	N/A	-	N/A	-	-	160.28	2.34	-	-
Number of point estimates	1	0	1	0	0	4	6	0	0
Number of studies	1^19^	0	1^43^	0	0	2^48^^,^^49^	1^92^	0	0
***Provider economic cost per death averted***									
Median/min-max/point estimate	1281.99	767.60-913.30	-	271.13	-	3496.26	-	-	39 628.04
Interquartile range (IQR)	998.24	N/A	-	73.84	-	N/A	-	-	N/A
Number of point estimates	16	2	0	4	0	1	0	0	1
Number of studies	5^14^^,^^20^^,^^37^, ^38^, ^39^‡	1^37^	0	1^56^	0	1^48^	0	0	1^100^
***Societal economic cost per death averted***									
Median/min-max/point estimate	7214.30	-	2412.17	-	-	10 449.50	3025.10	-	-
Interquartile range (IQR)	N/A	-	N/A	-	-	N/A	N/A	-	-
Number of point estimates	1	0	1	0	0	1	1	0	0
Number of studies	1^21^^,^^29^	0	1^43^	0	0	1^48^	1^89^	0	0
***Provider economic cost per DALY averted***									
Median/min-max/point estimate	44.51	23.22-27.09	-	10.41	-	-	13.5	124.38-623.75	974.46
Interquartile range (IQR)	35.04	N/A	-	13.87	-	-	N/A	N/A	N/A
Number of point estimates	14	2	0	14	0	0	1	2	1
Number of studies	3^14^^,^^20^^,^^38^^,^^39^‡	1^37^	0	2^55^^,^^56^	0	0	1^88^	1^102^	1^100^
*Societal economic cost per DALY averted*									
Median/min-max/point estimate	-	-	46.87	-	1.38-50.17	-	105.59	-	-
Interquartile range (IQR)	-	-	N/A	-	N/A	-	N/A	-	-
Number of point estimates	0	0	1	0	2	0	1	0	0
Number of studies	0	0	1^43^	0	2^52^^,^^54^	0	1^89^	0	0

Summary statistics are presented when there were enough data (refer to number of studies for given category); otherwise point estimate or range are presented. N/A indicates not applicable; –, no data; ±, all data for uncomplicated case treatment; ITN, insecticide-treated net; IRS, indoor residual spraying; IPTi, intermittent preventive treatment for infants; IPTp, intermittent preventive treatment for pregnant women; SMC, seasonal malaria chemoprevention.

∗ Study of ITN hammock.

† Savings in treatment intervention using ACT compared with monotherapies^91^

‡ Two references cover the same study^37^^,^^39^

### Chemoprevention

Chemoprevention is used for preventive treatment using antimalarial medicines and aims to prevent malarial illness by maintaining therapeutic drug levels in the blood throughout the period of greatest risk.

Eligible studies concerned seasonal chemoprevention in children (SMC) (n = 5, 42%)^46^, ^47^, ^48^, ^49^, ^50^ and intermittent preventive treatment in pregnant women (IPTp) (n = 4, 33%)^48^^,^^51^, ^52^, ^53^ and in infants (IPTi) (n = 3, 25%).^47^^,^^50^^,^^54^ Cost-effectiveness was explored by 2 studies each on SMC,^48^^,^^49^ IPTp,^52^^,^^54^ and IPTi^55^^,^^56^ (see Appendix 7 in Supplemental Materials found at https://doi.org/10.1016/j.jval.2021.01.013).

Chemoprevention eligible studies were all published after 2009.

#### Seasonal chemoprevention in children (SMC)

SMC is recommended by the WHO for all children <6 years during each transmission season in areas with highly seasonal malaria transmission in the sub-Sahel region of Africa. A SMC course was defined as the first dose, given under observation, of each treatment round. All studies had 3 or 4 treatment rounds, with one also evaluating 6 rounds.^49^ SMC studies analyzed the delivery costs of using community health workers (CHWs),^46^^,^^47^^,^^50^ volunteers,^48^^,^^49^ and/or mobile clinics^46^^,^^47^ or static facilities.^47^

#### Intermittent preventive treatment in pregnant women (IPTp)

IPTp is recommended for all women in their first or second pregnancy as part of antenatal care in malaria-endemic areas in Africa. IPTp studies took place at antenatal clinics^52^^,^^54^^,^^57^ or in the community.^52^^,^^53^

#### Intermittent preventive treatment of malaria in infants (IPTi)

IPTI is recommended for infants (<1 year of age) at the time of the second and third rounds of vaccination against DTP and against measles in areas of moderate to high malaria transmission in Africa. IPTi studies examined delivery at public health facilities and/or EPI/mobile clinics.

#### Unit cost and cost-effectiveness of chemoprevention

From a provider perspective, the median economic cost per child receiving a SMC course was $5.97 (IQR 6.79),^46^, ^47^, ^48^, ^49^, ^50^ and from a societal perspective in Ghana it was $71.^39^^,^^48^ Training, supervision, and distribution were the largest cost categories (see Appendix 8 in Supplemental Materials found at https://doi.org/10.1016/j.jval.2021.01.013).^46^, ^47^, ^48^, ^49^ The average cost per IPT dose administered to a pregnant woman was estimated at $0.86 (95% CI 0.58-1.22), including drug and personnel cost only.^57^ The societal cost per course of 2 IPTp doses ranged between $3.02 and $3.31 depending on the delivery platform, with distribution and user costs reported as main cost categories^52^ (see Appendix 7 in Supplemental Materials found at https://doi.org/10.1016/j.jval.2021.01.013). For IPTi, the median economic cost per infant protected (having received 3 doses) was $0.53 (IQR 0.59) ^51^^,^^55^^,^^56^ with drug cost, training, and IEC as the main unit cost categories (see Appendix 8 in Supplemental Materials found at https://doi.org/10.1016/j.jval.2021.01.013).

The cost-effectiveness of SMC was examined in Ghanaian settings only: the median cost per episode averted using different antimalarial drug regimens at community level was $121.50 (IQR 121.81) from a provider perspective and $177.34 (IQR 160.28) from a societal perspective.^48^^,^^49^ The cost per death averted with SMC was $3496 and $10 450 from a provider and societal perspective, respectively^48^ (Appendix 7 in Supplemental Materials found at https://doi.org/10.1016/j.jval.2021.01.013 and Table 1). Two studies examined the cost-effectiveness of IPTp, both from the societal perspective,^52^^,^^54^ with a median cost of $25.78 per DALY averted (Appendix 7 in Supplemental Materials found at https://doi.org/10.1016/j.jval.2021.01.013 and Table 1). Finally, depending on the drug regimen used, study location and transmission seasonality, IPTi cost-effectiveness from a provider perspective ranged between $0.86 and $22.46 per malaria episode averted,^55^^,^^56^ $125.25 and $376.38 per death averted,^56^ and $3.51 and $47.95 per DALY averted^55^^,^^56^ (Appendix 7 in Supplemental Materials found at https://doi.org/10.1016/j.jval.2021.01.013 and Table 1), largely driven by the very low unit cost of delivering the intervention.

### Diagnosis

All suspected malaria cases should be confirmed with a parasitological test, including RDT or microscopy. Diagnostic eligible studies examined RDT (17, 94%)^58^, ^59^, ^60^, ^61^, ^62^, ^63^, ^64^, ^65^, ^66^, ^67^, ^68^, ^69^, ^70^, ^71^, ^72^, ^73^, ^74^ and/or microscopy (n = 8, 50%)^58^, ^59^, ^60^, ^61^, ^62^^,^^65^^,^^69^^,^^70^^,^^75^ delivered to all-age presumptive malaria cases,^58^, ^59^, ^60^, ^61^, ^62^, ^63^, ^64^, ^65^, ^66^, ^67^, ^68^^,^^70^^,^^71^^,^^73^, ^74^, ^75^ children under 5 years of age with fever,^72^ or pregnant women^69^ at health facilities,^58^, ^59^, ^60^, ^61^, ^62^, ^63^^,^^65^^,^^67^, ^68^, ^69^, ^70^^,^^72^^,^^74^^,^^75^ drug shops,^64^ or in the community^66^^,^^71^^,^^73^ (see Appendix 9 in Supplemental Materials found at https://doi.org/10.1016/j.jval.2021.01.013).

#### Unit cost of diagnosis

Most studies analyzed the cost per case diagnosed and/or the cost per case diagnosed and treated, while others also examined the cost per additional case diagnosed and treated with RDT or microscopy compared to presumptive diagnosis (n = 9) and/or the cost per additional case diagnosed and/or treated with RDT/microscopy compared to microscopy/RDT (n = 3) (Appendix 9). Four studies estimated RDT unit cost for different malaria transmission risk^59^^,^^65^^,^^66^^,^^68^ or prevalence^71^ levels. (Appendix 9). From a provider perspective, the median economic cost per case diagnosed with RDT was $6.06 (IQR 6.23)^59^, ^60^, ^61^^,^^65^^,^^69^^,^^72^ and with microscopy $2.53 (IQR 5.24)^59^, ^60^, ^61^^,^^65^^,^^69^ The main cost categories included personnel, commodities, and supplies (see Appendix 10 in Supplemental Materials found at https://doi.org/10.1016/j.jval.2021.01.013).

### Treatment

Five parasite species cause malaria in humans: *Plasmodium falciparum, Plasmodium vivax, Plasmodium malariae, Plasmodium ovale,* and *Plasmodium knowlesi.* The first 2 pose the greatest health threat.^1^ A patient with uncomplicated malaria is a patient who presents with symptoms of malaria and a positive parasitological test but with no features of severe malaria. Uncomplicated malaria is assumed to be treated by outpatient health facility services or at the community level. Severe malaria is generally treated by inpatient healthcare services. Eligible treatment studies related to malaria treatment in children and adults (except pregnant women in their first trimester) with uncomplicated malaria or with severe malaria.

Studies examined the cost of treating *Plasmodium falciparum* malaria (n = 18, 86%)^76^, ^77^, ^78^, ^79^, ^80^, ^81^, ^82^, ^83^, ^84^, ^85^, ^86^, ^87^, ^88^, ^89^, ^90^, ^91^, ^92^, ^93^ or both *Plasmodium falciparum* and *Plasmodium vivax*^94^, ^95^, ^96^ (Appendix 9). Cost-effectiveness was analyzed in 5 studies.^88^^,^^89^^,^^92^^,^^95^^,^^96^ Most studies were published after 2010^76^^,^^78^^,^^80^, ^81^, ^82^, ^83^, ^84^, ^85^, ^86^, ^87^, ^88^, ^89^, ^90^^,^^94^^,^^96^ and concerned uncomplicated malaria only,^78^^,^^80^^,^^82^, ^83^, ^84^^,^^88^^,^^89^^,^^91^, ^92^, ^93^^,^^95^^,^^96^ both uncomplicated and severe malaria,^76^^,^^79^^,^^81^^,^^86^^,^^90^^,^^94^ with severe or moderate anemia^81^ or severe malaria only^77^^,^^85^^,^^87^ in all ages,^78^^,^^80^^,^^82^^,^^83^^,^^89^^,^^91^^,^^93^ infants/children,^76^^,^^80^^,^^82^^,^^83^^,^^85^^,^^87^, ^88^, ^89^, ^90^^,^^92^^,^^95^^,^^96^ or pregnant or postpartum women^94^ (Appendix 9). Studies examined the cost of treatment at health facilities^76^, ^77^, ^78^, ^79^^,^^81^^,^^84^, ^85^, ^86^, ^87^, ^88^^,^^90^^,^^92^^,^^94^, ^95^, ^96^ or in the community,^79^^,^^80^^,^^82^^,^^83^^,^^89^^,^^91^^,^^93^ at times in the context of integrated community case management.^80^^,^^82^^,^^83^ Cost data were commonly reported per uncomplicated and/or severe case treated, occasionally per case diagnosed and treated.^83^^,^^92^ A few studies also examined the incremental cost or cost-effectiveness of different antimalarial drugs^79^^,^^87^^,^^88^^,^^92^^,^^95^^,^^96^ or delivery platforms^78^^,^^89^ (Appendix 9).

#### Unit cost and cost-effectiveness of treatment

From a provider perspective, the median economic cost was $9.31 (IQR 8.90) per uncomplicated case treated;^76^^,^^78^^,^^79^^,^^82^^,^^84^^,^^88^^,^^90^^,^^94^ $7.15 (IQR 2.77) per uncomplicated case diagnosed and treated^78^^,^^83^^,^^92^ and $89.93 (IQR 51.10) per severe case treated (Table 1).^76^^,^^77^^,^^79^^,^^88^^,^^90^^,^^94^ From a societal perspective, it was $11.90 (IQR 11.40) per uncomplicated case diagnosed and treated^83^^,^^90^^,^^92^ and $145.23 (IQR 118.88) per severe case treated.^77^^,^^90^ The largest cost categories were personnel and drugs (see Appendix 11 in Supplemental Materials found at https://doi.org/10.1016/j.jval.2021.01.013).

The cost-effectiveness of treating uncomplicated malaria using dihydroartemisinin-piperaquine compared to no treatment was US$13.50 per DALY averted from a provider perspective in an urban district hospital of Tanzania^88^ (Appendix 9). In another district hospital of Tanzania, the median provider cost-effectiveness of artemisinin combination therapy compared to monotherapy was $0.30 (IQR 1.47) per case averted and resulted in societal savings of $30.99 (IQR 2.34) per case averted.^92^ In rural Ghana, the societal cost-effectiveness of treating under-5 fevers at home using community health workers compared to routine treatment seeking was $3025 per death averted and $106 per DALY averted^89^ (Appendix 9).

### Surveillance

Surveillance is “the continuous and systemic collection, analysis and interpretation of disease specific data, and the use of that data in the planning, implementation and evaluation of public health practice.”^97^ In settings in which transmission is high, surveillance is often integrated into broader routine health information systems; where transmission is low and malaria is being eliminated, surveillance is used to identify, investigate, and eliminate foci of continuing transmission, prevent and cure infections, and confirm elimination. Surveillance studies concerned active case detection (n = 5),^98^, ^99^, ^100^, ^101^, ^102^ surveillance systems for malaria epidemics (n = 2),^103^^,^^104^ and entomological surveillance (n = 2).^105^^,^^106^ Given the specificity of each surveillance intervention type, unit cost and cost-effectiveness data are presently separately for each intervention.

#### Active case detection (ACD)

ACD is used to detect symptomatic cases that are not detected by passive case detection (ie, when a person seeks care) and asymptomatic cases in the community. ACD is generally conducted intermittently outside health facilities by health workers who visit patients at their houses, or elsewhere, and involves administering a parasitological diagnosis of everyone in a targeted population, immediate treatment to positive cases, and follow-up to ensure complete cure.^97^ Proactive case detection (pACD) is undertaken in populations that have limited access to facilities or inadequate health-seeking behavior and in high-risk groups. Re-active case detection (rACD) is undertaken in response to an index case (usually seen at a health facility), the epidemiological characteristics of which trigger additional ACD, whereby a household or a population potentially linked to the index case is tested or screened for symptoms and tested before treatment.^97^ ACD studies were all published after 2011 and related to rACD^100^^,^^102^ or pACD.^98^^,^^99^^,^^101^ The provider economic cost of rACD was $38.63 per person tested and $32.07 per case treated^100^ and for pACD $4.79 per person tested,^101^ $7.18 per person screened (ie, tested and, if positive, treated),^98^ and $37.87 per case treated.^101^ Various types of cost output measures were used, and there was not enough commonality across studies to review unit cost driver data (see Appendix 12 in Supplemental Materials found at https://doi.org/10.1016/j.jval.2021.01.013). The incremental cost-effectiveness of pACD was $79.25 per case averted, $39 628 per death averted, and $623.75 per DALY averted compared to no pro-ACD.^101^ No study examined the cost-effectiveness of rACD.

#### Surveillance systems for epidemics

Studies on surveillance systems for epidemics were published before 2010.^103^^,^^104^ The provider economic cost ranged from $0.04 per person per year to $1.47 per person protected and cost-effectiveness between $1.28 and $389.82 per case averted depending on transmission intensity (Appendix 12).^104^

#### Entomological surveillance

Entomological studies were published after 2011 and concerned the provider cost of community-based mosquito trapping schemes.^105^^,^^106^ Unit cost measures included the cost per person-night of sampling and the cost per specimen of Anopheles caught (Appendix 12). None of the studies provided data on the cost-effectiveness of these interventions using a health outcome measure.

### Combinations of Malaria Control Interventions

Eleven studies examined more than one type of intervention, and all were published on or after 2012, except one (see Appendix 13 in Supplemental Materials found at https://doi.org/10.1016/j.jval.2021.01.013).^107^, ^108^, ^109^, ^110^, ^111^, ^112^, ^113^, ^114^, ^115^, ^116^, ^117^ Studies investigated the cost or cost-effectiveness of combining preventive interventions only,^108^^,^^109^^,^^111^, ^112^, ^113^, ^114^^,^^116^ commonly vector control^108^^,^^113^^,^^114^^,^^116^ in sub-Saharan African settings, while others examined the unit cost of more comprehensive intervention packages in relatively low endemic or elimination settings^107^^,^^110^^,^^114^^,^^115^^,^^117^ (Fig. 2). These latter studies also analyzed the incremental cost-effectiveness of adding 1 or more interventions to routine activities.^107^^,^^117^ Given the variable focus of these studies and the different unit cost output and cost-effectiveness outcome measures, no summary statistics could be calculated.

## Discussion

This review identified 103 studies with primary data on the unit cost of delivering the currently WHO-recommended malaria control interventions, either individually or in combination, with approximately one third of the studies also providing evidence on the cost-effectiveness of these interventions. Summarizing the available evidence was a challenge given the high degree of heterogeneity within and across the studied interventions. Overall, cost-effectiveness studies reiterated the value for money of malaria control, although global efforts in methodological and reporting standards are required for the evidence to be useful outside their study contexts.^118^, ^119^, ^120^, ^121^, ^122^, ^123^ These results are important when considering how unit costs and cost-effectiveness data from one study are frequently used in different settings with limited or no adaptation.

The available evidence also concerned largely individual interventions and less so that of comprehensive packages of interventions, which is recommended for effective control.^4^ The number of studies of malaria control interventions in combination increased in recent years, although these studies appeared to be more common in lower-transmission settings and designed to assess the incremental cost-effectiveness of adding one intervention on top of another one. These studies shine little light on the change in efficiency of combining the delivery of interventions. The 2 studies that allowed for the comparison of delivering interventions separately or in combination^108^ with ITNs and IRS and with ITNs and IPTp^109^ suggest the cost savings of combining the delivery of both is minimal. It was difficult to compare the other packages of interventions within the studies themselves, or to the wider cost and cost-effectiveness literature due to the way the data were presented. As health systems move toward more integrated service delivery, it will be important that future cost and health outcome data allow for analyses of efficiency gains and economies of scope. Few studies examined malaria control interventions in the presence of comorbidities. For instance, only 1 study used anemia reduction as an outcome of malaria control despite the close link between the 2 conditions.^124^ Studies also rarely explicitly considered the quality of interventions or their equitable coverage when examining unit cost or/and cost-effectiveness.

Our systematic review has some limitations. Non-English search terms and conference abstracts or posters were excluded as well as modeling studies relying exclusively on secondary unit cost or cost-effectiveness data. Study heterogeneity, in terms of analytical perspective, type of cost, unit cost output, and/or cost-effectiveness comparator made it impossible to generate summary statistics for all interventions. Cost-effectiveness results are influenced by the coverage rate of the intervention under investigation, access to other control interventions, and wider health system characteristics. However, these details were rarely reported in the eligible studies. Most studies were conducted within trial contexts as opposed to routine delivery, which may distort unit cost, cost drivers, and cost-effectiveness results. Often, costing studies reported point estimates and not ranges, which likely reflect the real-world uncertainty associated with costing parameters. Such frustrations with costing and cost-effectiveness literature are not unique to malaria control interventions.^119^^,^^125^ Finally, a quality assessment or risk of bias assessment was originally planned as part of the study. Having reviewed and piloted various tools,^126^, ^127^, ^128^, ^129^, ^130^, ^131^ it became apparent that they were not suitable, and indeed led to misleading findings. The tools were designed for cost-effectiveness studies with both costs and outcome data primarily from trials as the default. Our review, however, contains both costing and cost-effectiveness studies, and the purely costing studies “scored” consistently lower given they did not cover all the domains. We, therefore, chose not to undertake a quality assessment using a tool that was not fit for the purpose, nor did we think it insightful to develop a bespoke tool for this study given issues of external validity.^131^ Deeper reflections on assessing the quality of these data is an important next step.

## Conclusions

To our knowledge, this is the first systematic review to identify and examine the unit cost and cost-effectiveness of all WHO-recommended interventions, implemented individually or in combinations. We identified 103 different costing studies, with nearly one third analyzing the cost-effectiveness of malaria control. The most commonly examined malaria control intervention was ITN, with 26 costing studies and 7 ITN cost-effectiveness analyses, followed by treatment with 21 costing and 5 cost-effectiveness studies. Tanzania, Zambia, and Ghana were by far the most common study settings. The number of studies on combinations of interventions increased recently, although most focused on lower-transmission settings or preventive interventions. Our results indicate that studies used more frequently a provider perspective with more limited societal considerations. Looking to the future, more standardized methods and reporting are needed, as well as guidance on options to transfer data across contexts.

## Article and Author Information

**Author Contributions:** *Concept and design*: Conteh, Shuford, Kolaczinski, Patouillard

*Acquisition of data*: Conteh, Shuford, Agboraw, Patouillard

*Analysis and interpretation of data*: Conteh, Shuford, Agboraw, Kont, Kolaczinski, Patouillard

*Drafting of the manuscript*: Conteh, Shuford, Kont, Patouillard

*Critical revision of the paper for important intellectual content*: Conteh, Shuford, Agboraw, Kont , Kolaczinski, Patouillard

*Statistical analysis*: Conteh, Patouillard

*Obtaining* *funding*: Conteh, Kolaczinski, Patouillard

*Administrative, technical, or logistic* *support*: Shuford, Patouillard

*Supervision*: Conteh, Patouillard

**Conflict of Interest Disclosures:** The authors reported no conflicts of interest.

**Funding/Support:** This work was supported by several grants from the 10.13039/100000200United States Agency for International Development and the 10.13039/100000865Bill and Melinda Gates Foundation.

**Role of the** **Funder/Sponsor:** The funder had no role in the design and conduct of the study; collection, management, analysis, and interpretation of the data; preparation, review, or approval of the manuscript; and decision to submit the manuscript for publication.
