# Supplementary material for: Costs and Cost-Effectiveness of Malaria Control Interventions: A Systematic Literature Review
Source: Value Health. 2021 Aug;24(8):1213–22. doi: 10.1016/j.jval.2021.01.013 (PMC8324482; doi:10.1016/j.jval.2021.01.013)
Supplement: Appendix 1 [file mmc1.pdf]

Appendix 1: Search strategies

We employed the following keywords (and MESH terms where relevant) and adapted the specific search strategy as required for each individual database. The search strategies draws on the pre-tested SIGN search filters which have been devised for running in Ovid implementations of Medline and Embase along with other more specialised databases.

Component	Key terms (1 AND 2)
1. Cost	cost* OR economic* OR financ*<br><br>*by comparison:*<br>*White: cost**<br>*DCP: cost* or economic**<br>*GHCC: cost* or economic or finance*<br>*Yukich: cost OR effective OR effectiveness OR benefit*
2. Malaria	malaria OR plasmodium<br><br>*by comparison:*<br>*White: malaria OR falciparum*<br>*DCP: malaria OR plasmodium OR falciparum*<br>*Yukich : malaria OR falciparum OR plasmodium*

Medline (OVID)
EMBASE (OVID)
Econlit
The National Health Service Economic Evaluation Database (NHS EED)
The Cost-effectiveness Analysis Registry
Cochrane library
Web of Science
Latin American and Caribbean Health Sciences Literature (LILACS)

Medline (OVID)

1. exp "Costs and Cost Analysis"/
2. (cost* or economic* or financ*).mp
3. 1 or 2
4. malaria/ or malaria, cerebral/ or malaria, falciparum/ or malaria, vivax/
5. plasmodium/ or plasmodium falciparum/ or plasmodium knowlesi/ or plasmodium malariae/ or

plasmodium ovale/ or plasmodium vivax/
6. (malaria or plasmodium).mp
7. 4 or 5 or 6
8. 3 and 7
9. limit 8 to yr="2005 -Current"

Embase (OVID)

1. malaria/ or cerebral malaria/ or malaria falciparum/ or plasmodium knowlesi malaria/ or plasmodium malariae infection/ or plasmodium ovale malaria/ or plasmodium vivax malaria/
2. plasmodium/ or plasmodium falciparum/ or plasmodium knowlesi/ or plasmodium malariae/ or plasmodium ovale/ or plasmodium vivax/
3. (malaria or plasmodium).mp
4. 1 or 2 or 3
5. exp economic evaluation/
6. (cost* or economic* or financ*).mp
7. 5 or 6
8. 4 and 7
9. limit 8 to yr="2005 -Current"

EconLit

Search History

#	Query	Limiters/Expanders	Last Run Via	Results
S3	S1 AND S2	Search modes - Boolean/Phrase	Interface - EBSCOhost Research Databases<br>Search Screen - Advanced Search<br>Database - EconLit with Full Text	357
S2	malaria OR plasmodium	Limiters - Published Date: 20050101-20181231<br>Search modes - Boolean/Phrase	Interface - EBSCOhost Research Databases<br>Search Screen - Advanced Search<br>Database - EconLit with Full Text	387
S1	cost* OR economic* OR financ*	Limiters - Published Date: 20050101-20181231<br>Search modes - Boolean/Phrase	Interface - EBSCOhost Research Databases<br>Search Screen - Advanced Search<br>Database - EconLit with Full Text	680,933

Cochrane Library

#1 MeSH descriptor: [Costs and Cost Analysis] explode all trees
#2 cost* or economic* or financ*
#3 #1 or #2
#4 MeSH descriptor: [Malaria] this term only
#5 MeSH descriptor: [Malaria, Vivax] this term only
#6 MeSH descriptor: [Malaria, Falciparum] this term only
#7 MeSH descriptor: [Malaria, Cerebral] this term only
#8 MeSH descriptor: [Plasmodium] this term only
#9 MeSH descriptor: [Plasmodium falciparum] this term only

#10 MeSH descriptor: [Plasmodium knowlesi] this term only
#11 MeSH descriptor: [Plasmodium malariae] this term only
#12 MeSH descriptor: [Plasmodium ovale] this term only
#13 MeSH descriptor: [Plasmodium vivax] this term only
#14 malaria or plasmodium
#15 #4 or #5 or #6 or #7 or #8 or #9 or #10 or #11 or #12 or #13 or #14
#16 #3 and #15 Publication Year from 2005 to 2018

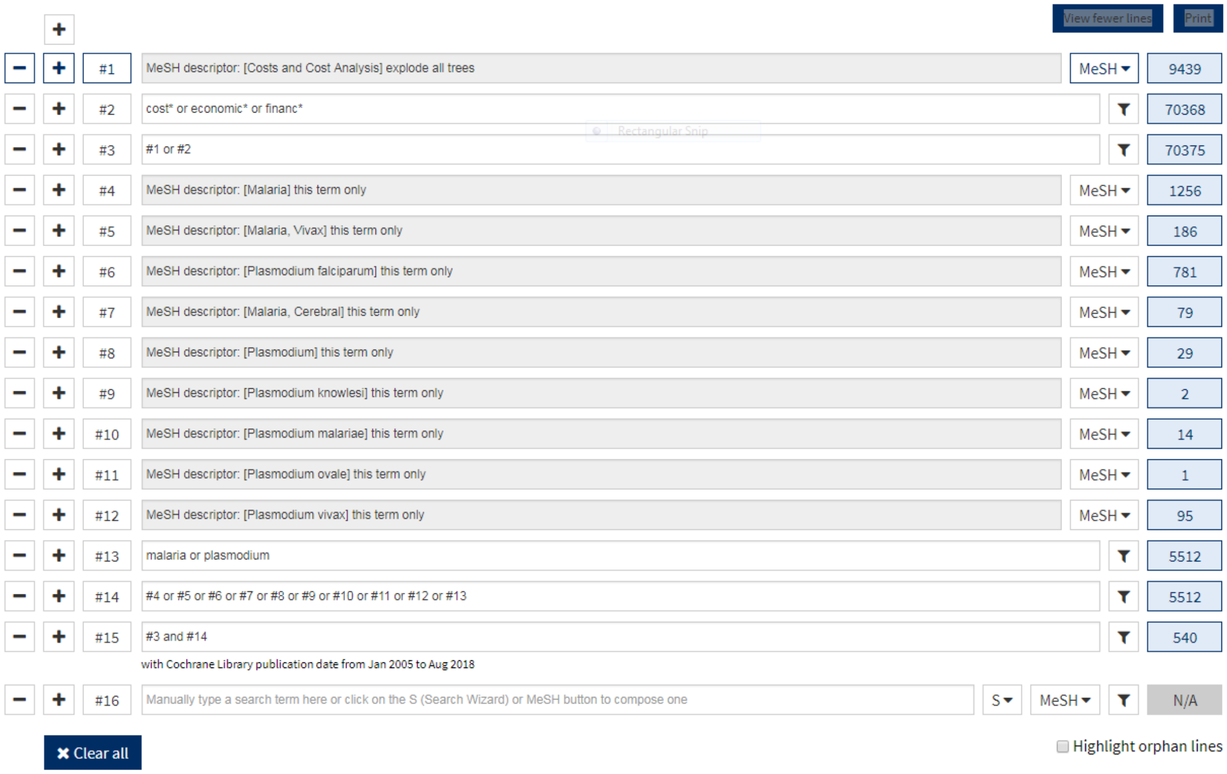

NHS EED

These are covered by Cochrane Library (separate search for verification check below)
1 - (cost*) OR (economic*) OR (financ*) IN NHSEED FROM 2005 TO 2018 = 11644
2 - (malaria) OR (plasmodium) IN NHSEED FROM 2005 TO 2018 = 85
3 - #1 AND #2 = 85

Web of Science

# 1
TS=(cost* OR economic* OR financ*)
Indexes=SCI-EXPANDED, SSCI, A&HCI, CPCI-S, CPCI-SSH, ESCI Timespan=2005-2018

# 2
TS=(malaria OR plasmodium)

Indexes=SCI-EXPANDED, SSCI, A&HCI, CPCI-S, CPCI-SSH, ESCI Timespan=2005-2018

3
#2 AND #1
Indexes=SCI-EXPANDED, SSCI, A&HCI, CPCI-S, CPCI-SSH, ESCI Timespan=2005-2018

LILACS

(cost* OR economic* OR financ*) AND (malari* OR plasmodium) AND (instance:"regional") AND (db:("LILACS") AND year_cluster:("2011" OR "2007" OR "2012" OR "2014" OR "2008" OR "2013" OR "2009" OR "2010" OR "2015" OR "2005"))
