# Supplementary material for: Costs and Cost-Effectiveness of Malaria Control Interventions: A Systematic Literature Review
Source: Value Health. 2021 Aug;24(8):1213–22. doi: 10.1016/j.jval.2021.01.013 (PMC8324482; doi:10.1016/j.jval.2021.01.013)
Supplement: Appendix 2 [file mmc2.pdf]

Appendix 2: Distribution of eligible studies by intervention and year

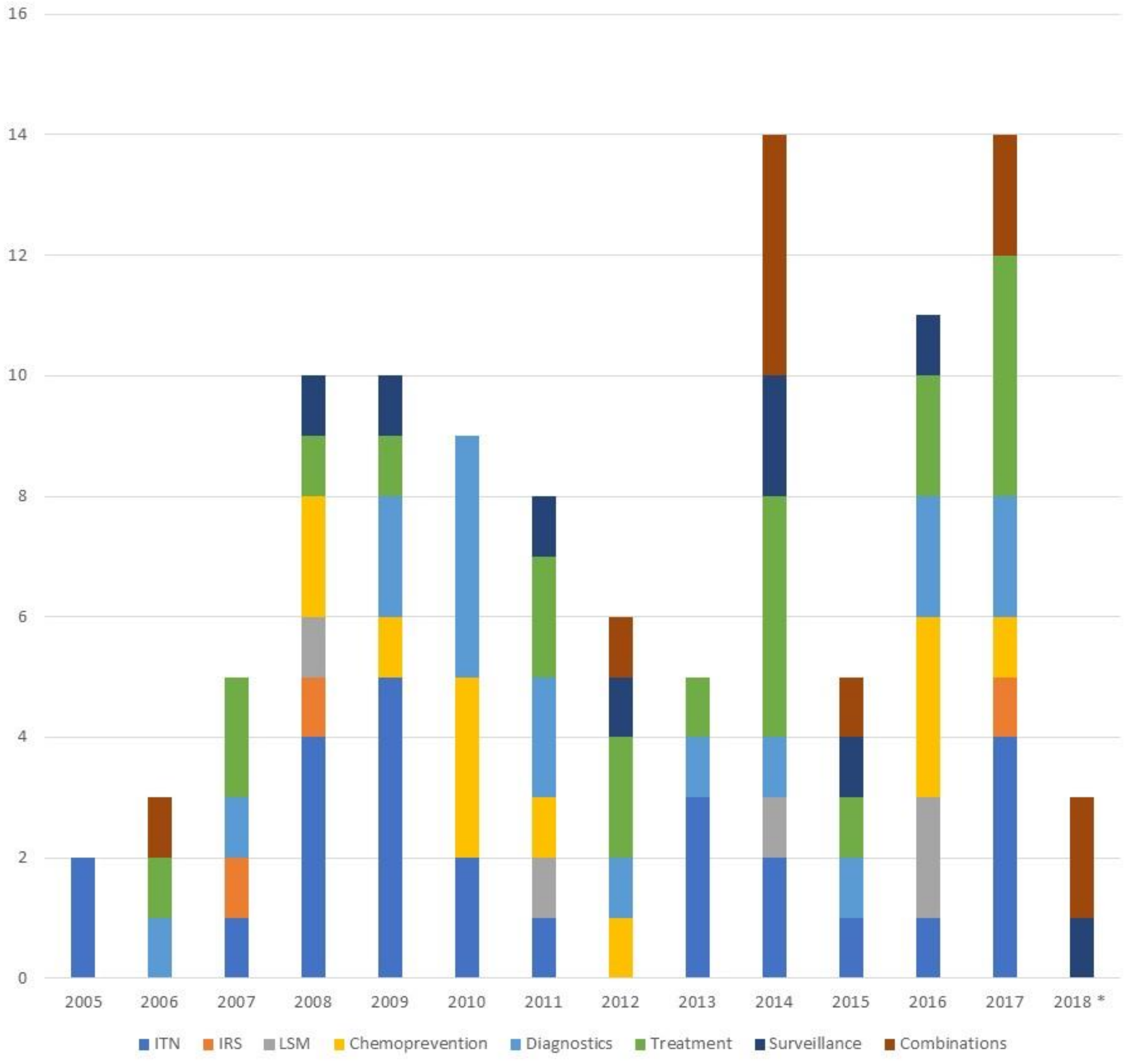

Legend: year 2018 goes up to 31 August.

ITN: insecticide-treated net; IRS: indoor residual spraying; LSM: larval source management.
