# Supplementary material for: Costs and Cost-Effectiveness of Malaria Control Interventions: A Systematic Literature Review
Source: Value Health. 2021 Aug;24(8):1213–22. doi: 10.1016/j.jval.2021.01.013 (PMC8324482; doi:10.1016/j.jval.2021.01.013)
Supplement: Appendix 3 [file mmc3.pdf]

Appendix 3: Characteristics and results of eligible studies considering vector control interventions

	Country	Setting	Delivery strategy (platform)	Population group targeted	Commodities distributed, or areas treated	Perspective	Cost type	Unit cost or CE estimate (US$ 2018)	Output or health outcome measure
Insecticide treated nets (ITN)									
Becker-Dreps et al (2009)[14]	DRC	Urban	Continuous (ANC)	Pregnant women	0.02 million LLIN	Provider	Economic	12.28	per net distributed
						Provider	Economic	530.36	per infant death averted compared to standard ANC care
						Provider	Economic	20.25	per year of life saved compared to standard ANC care
						Provider	Economic	22.21	per DALY averted compared to standard ANC care
Bonner K et al (2011)[15]	United Republic of Tanzania (Mainland)	Mixed	Campaign	Children under five years of age	9 million LLIN	Provider	Financial	9.12	per net distributed
De Allegri et al (2010)[16]	Burkina Faso	Mixed	Continuous (ANC, social marketing)	Pregnant women; All	0.01-0.05 million LLIN	Provider	Financial	9.01	per net distributed (ANC)
						Provider	Financial	10.10	per net distributed (social marketing)
						Provider	Economic	6.01	per net distributed (all platforms)
Grabowsky et al (2005)[17]	Zambia	Rural	Campaign	Children during measles vaccination	0.07 million ITN with retreatment	Provider	Financial	6.35	per net distributed
Kolaczinski et al (2010)[18]	Uganda	Urban	Campaign<br>Continuous (ANC)	Households with pregnant women or children under five; Pregnant women	0.01-0.02 million LLIN	Provider	Financial	7.49-8.57	per net distributed across strategies
						Provider	Economic	1.16-1.77	per treated net year across strategies
						Provider	Economic	3.48-5.31	per net distributed across strategies
Morel et al (2013)[19]	Vietnam	NR - mountainous forested area	NR	Population aged ten years old or more	NR LLIN hammock	Provider	Financial	12.82	per net distributed
						Societal	economic	137.34	per case averted compared to routine ITN

						Provider	economic	152.60	per episode averted compared to routine ITN
Mueller et al (2008)[20]	Togo	Mixed	Campaign	Children 9 months to 5 years during measles vaccination	0.9 million LLIN	Provider	Economic	5.87	per net distributed
						Provider	Economic	4.34	per case averted compared to no ITN during campaign
						Provider	Economic	1138.48	per death averted compared to no ITN during campaign
						Provider	Economic	21.80	per DALY averted compared to no ITN during campaign
Mulligan et al (2008)[21]	United Republic of Tanzania (Mainland)	Mixed	Continuous (vouchers at ANC)	Pregnant women and their infants	1.4 million ITN with retreatment	Provider	Financial	10.61	per net distributed
						Provider	Economic	9.46	per net distributed
						Provider	Financial	5.29	per treated net year
						Provider	Economic	4.84	per treated net year (excluding start-up costs)
						Provider	Economic	16.25	per case averted compared to no ITN
						Provider	Economic	1091.25	per death averted compared to no ITN
Ntuku et al (2017)[22]	DRC	NR	Campaign (health facilities, households)	NR	0.6-3 million LLIN	Provider	Financial	6.97-7.01	per net distributed across strategies
Pemba et al (2008)[23]	Malawi	Rural	Campaign	All	15 households	Provider	NR	27.57	per household ITN use assuming 5 nets per household
Renggli et al (2013)[24]	United Republic of Tanzania (Mainland)	NR	Campaign	All	18 million LLIN	Provider	Financial	5.90	per net distributed
Scates et al (2016)[25]	Mali	Rural, urban, Mixed	Continuous (public health facilities) Campaign	Pregnant women, children under 5; All	1-1.8 million LLIN	Provider	Financial	2.19	per net targeted
						Provider	Economic	2.29	per net targeted
						Provider	Financial	4.40	per net distributed
						Provider	Economic	4.59	per net distributed
						Provider	Economic	1.53	per treated net year
						Provider	Economic	0.77	per person-year protected
Scates et al (2017a)[26]	United Republic of Tanzania (Mainland)	Rural	Continuous (schools)	Primary school children	0.5 million LLIN	Provider	Financial	4.66	per net targeted
						Provider	Economic	4.77	per net targeted
						Provider	Financial	9.31	per net distributed

						Provider	Economic	9.53	per net distributed
						Provider	Economic	3.18	per treated net year
						Provider	Economic	1.59	per person-year protected
Scates et al (2017b)[27]	United Republic of Tanzania (Mainland)	Rural	Continuous (schools)	Primary school children	0.3 million LLIN	Provider	Financial	1.80	per net targeted
						Provider	Economic	1.87	per net targeted
						Provider	Financial	3.60	per net distributed
						Provider	Economic	3.74	per net distributed
						Provider	Economic	1.25	per treated net year
						Provider	Economic	0.62	per person-year protected
Sedlmayr et al (2013)[28]	Zambia	Rural	Campaign	Cotton farmers	0.04 million LLIN	Provider	NR	5.71	per net distributed
Smith Paintain et al (2014)[29]	Ghana	Mixed	Campaign	All	12.5 million LLIN	Societal	Financial	7.41	per net distributed
						Societal	Economic	3.16	per net distributed
						Provider	Financial	7.10	per net distributed
						Provider	Economic	2.93	per net distributed
						Societal	Economic	7214.30	per additional death averted compared to. no campaign
Stevens et al (2005)[30]	Malawi	Mixed	Continuous (public health facilities, community, social marketing)	All; Pregnant women, children under five	1.5 million ITN with retreatment	Provider	Economic	3.97	per net distributed
						Provider	Economic	6.66	per treated net year
WHO (2009)[31]	United Republic of Tanzania (Zanzibar)	Rural, urban, Mixed	Continuous (ANC)	Pregnant women, children under five, All	0.02-0.2 million LLIN	Provider	Financial	9.01-9.94	per net delivered across population groups
						Provider	Economic	4.05-4.68	per net delivered across population groups
WHO (2009)[32]	Uganda	Mixed	Campaign	Pregnant women, children under five	1.7 -4.2 million LLIN	Provider	Financial	8.04-8.29	per net delivered across funding sources
						Provider	Economic	3.75-3.78	per net delivered across funding sources
WHO (2009)[33]	Kenya	Mixed	Continuous (ANC, social marketing)	Pregnant women, children under five; All	0.07-1.8 million ITN with retreatment and LLIN	Provider	Financial	6.81-10.09	per net delivered across types of nets and urbanicity
						Provider	Economic	4.46-6.07	per net delivered across types of nets and urbanicity
Wisniewski et al (2017)[34]	United Republic of Tanzania (Mainland)	Rural	Continuous (ANC, EPI)	Pregnant women, children under five	0.8 million LLIN	Provider	Financial	3.94	per net targeted

						Provider	Economic	4.17	per net targeted
						Provider	Financial	7.88	per net distributed
						Provider	Economic	8.35	per net distributed
						Provider	Economic	2.78	per treated net year
						Provider	Economic	1.39	per person-year protected
Yukich et al (2007) *[37,39]	Eritrea, Togo, Malawi, Senegal, United Republic of Tanzania (Mainland)	Mixed	Continuous (ANC, health facilities, CHW, social marketing), Campaign (vaccination)	Pregnant women, All; Children	0.7- 6.4 million ITN with retreatment	Provider	Financial	5.33-13.34	per net distributed across settings
						Provider	Economic	4.17-10.38	per net distributed across settings
						Provider	Financial	3.64-9.03	per treated net year across settings
						Provider	Economic	2.75-7.80	per treated net year across settings
						Provider	Economic	1514.46-3774.54	per death averted compared to no ITN across settings
						Provider	Economic	46.44-114.81	per DALY averted compared to no ITN across settings
Yukich et al (2009)[38]	Eritrea	Mixed	Continuous (ANC, maternal and child health clinics, community, local administrations); Campaign retreatment at health facilities/community	Women; All	0.9 million ITN with retreatment	Provider	Financial	6.09	per net distributed
						Provider	Economic	5.13	per net distributed
						Provider	Financial	1.84	per treated net year
						Provider	Economic	1.56	per treated net year
						Provider	Economic	565.02	per death averted compared to no ITN using cost per TNY)-
						Provider	Economic	1869.21	per death averted compared to no ITN (using cost per ITN)
						Provider	Economic	16.77	per DALY averted compared to no ITN (using cost per TNY)
						Provider	Economic	56.76	per DALY averted compared to no ITN (using cost per ITN)
Yukich et al (2014)[35]	Ghana	Rural, urban, Mixed	Continuous (ANC, EPI, schools)	Pregnant women, children under-five, primary school children	0.6-0.9 million LLIN	Provider	Financial	3.93-4.20	per net targeted (ANC, EPI)
						Provider	Financial	2.13	per net targeted(schools)
						Provider	Economic	4.19-4.50;	per net targeted (ANC, EPI)

						Provider	Economic	2.26	per net targeted (schools)
						Provider	Financial	7.86-8.40	per net distributed (ANC, EPI)
						Provider	Financial	4.27	per net distributed (schools)
						Provider	Economic	8.39-9.02	per net distributed (ANC, EPI)
						Provider	Economic	4.53	per net distributed (schools)
						Provider	Economic	2.79-3.00	per treated net year (ANC, EPI)
						Provider	Economic	1.51	per treated net year (schools)
						Provider	Economic	1.40-1.50	per person-year protected (ANC, EPI)
						Provider	Economic	0.76	per person-year protected (schools)
Yukich et al (2016)[36]	United Republic of Tanzania (Zanzibar)	Rural	Continuous (ANC, community, EPI)	Pregnant women, children, eligible community members	0.2 million LLIN	Provider	Financial	4.38	per net targeted
							Economic	5.20	per net targeted
							Financial	8.76	per net distributed
							Economic	10.40	per net distributed
							Economic	3.47	per treated net year
							Economic	1.73	per person-year protected
Indoor Residual Spraying (IRS)									
Cico et al (2018)[40]	12 African countries**	Mixed	Campaign	All	156,362-738,810 structures (38-158 m^2 /structure) sprayed with organophosphate only insecticide or organophosphate and pirimiphos-methyl	Provider	Economic	14.75-41.75	per 100m2 sprayed across settings
						Provider	Economic	3.40-9.10	per person protected across settings

						Provider	Economic	12.64-29.55	per structure sprayed across settings
Pemba et al (2008)[23]	Malawi	Rural	Trial aligned to usual district activities	All	15 households with pyrethroid	Provider	Financial	2.76	per structure sprayed
Yukich et al (2007)[37]	South Africa, Mozambique	Mixed	Campaign	All	150,000-300,000 structures sprayed with dichlorodiphenyltrichloroe-thane(DDT), pyrethroids, carbamates, organophosphates	Provider	Economic	4.22-5.03	per person protected
						Provider	Financial	11.74	per structure sprayed
						Provider	Economic	9.60	per structure sprayed
						Provider	Financial	5073.57-5620.53	per death averted compared to no IRS
						Provider	Economic	767.55-913.32	per death averted compared to no IRS
						Provider	Financial	153.51-170.28	per DALY averted compared to no IRS
						Provider	Economic	23.22-27.09	per DALY averted compared to no IRS
Larviciding									
Dambach et al (2016)[41]	Burkina Faso	Rural	Campaign	All	240 hectares	Provider	Financial	0.83-1.13	per person protected depending on spraying coverage
Kusumawathie et al (2008)[42]	Sri Lanka	NR	Campaign	All living in outbreak areas	Two dams			13.70	per m^2 sprayed
Maheu-Giroux et al (2014)[43]	Republic of Tanzania	Urban	NR - microbial	All	56 km2	Provider	Economic	0.95	per person-year protected
						Provider	Financial	3.92	per case averted
						Societal	Economic	2.62	per case averted
						Provider	Financial	3646.05	per death averted
						Societal	Economic	2412.17	per death averted
						Provider	Financial	70.85	per DALY averted compared to no larviciding in a scenario of high transmission or no other control intervention

						Societal	Economic	46.87	per DALY averted compared to no larviciding in a scenario of high transmission or no other control intervention
Rahman et al. (2016)[44]	Tanzania	Rural	Campaign - microbial	All	6873 breeding sites	Provider	Financial	1.22	per person protected per year
						Provider	Economic	1.53	per person protected per year
Worrall et al. (2011) [45]	Republic of Tanzania, Kenya	Mixed	Campaign	All	58-563 km^2	Provider	Financial	1.06-1.85	per person protected per year
						Provider	Economic	0.99-1.88	per person protected per year

Note: ITN: insecticide treated bed nets; LLIN: long lasting insecticidal treated bed nets, IRS: indoor residual spraying;*single study with results published in two papers; CE: cost-effectiveness; **includes Benin, Ethiopia, Ghana, Kenya, Madagascar, Mali, Mozambique, Rwanda, Senegal, Tanzania, Zambia, Zimbabwe; NR: not reported by the study; ANC: antenatal care clinic; DALY: disability adjusted life year; Mixed refers to rural and urban study settings .
