# Supplementary material for: Costs and Cost-Effectiveness of Malaria Control Interventions: A Systematic Literature Review
Source: Value Health. 2021 Aug;24(8):1213–22. doi: 10.1016/j.jval.2021.01.013 (PMC8324482; doi:10.1016/j.jval.2021.01.013)

Appendix 6: Breakdown of economic cost per person protected with LSM, by cost centre and study country focus (constant 2018 US$)
When no breakdown available, unit cost data represented using a single colour

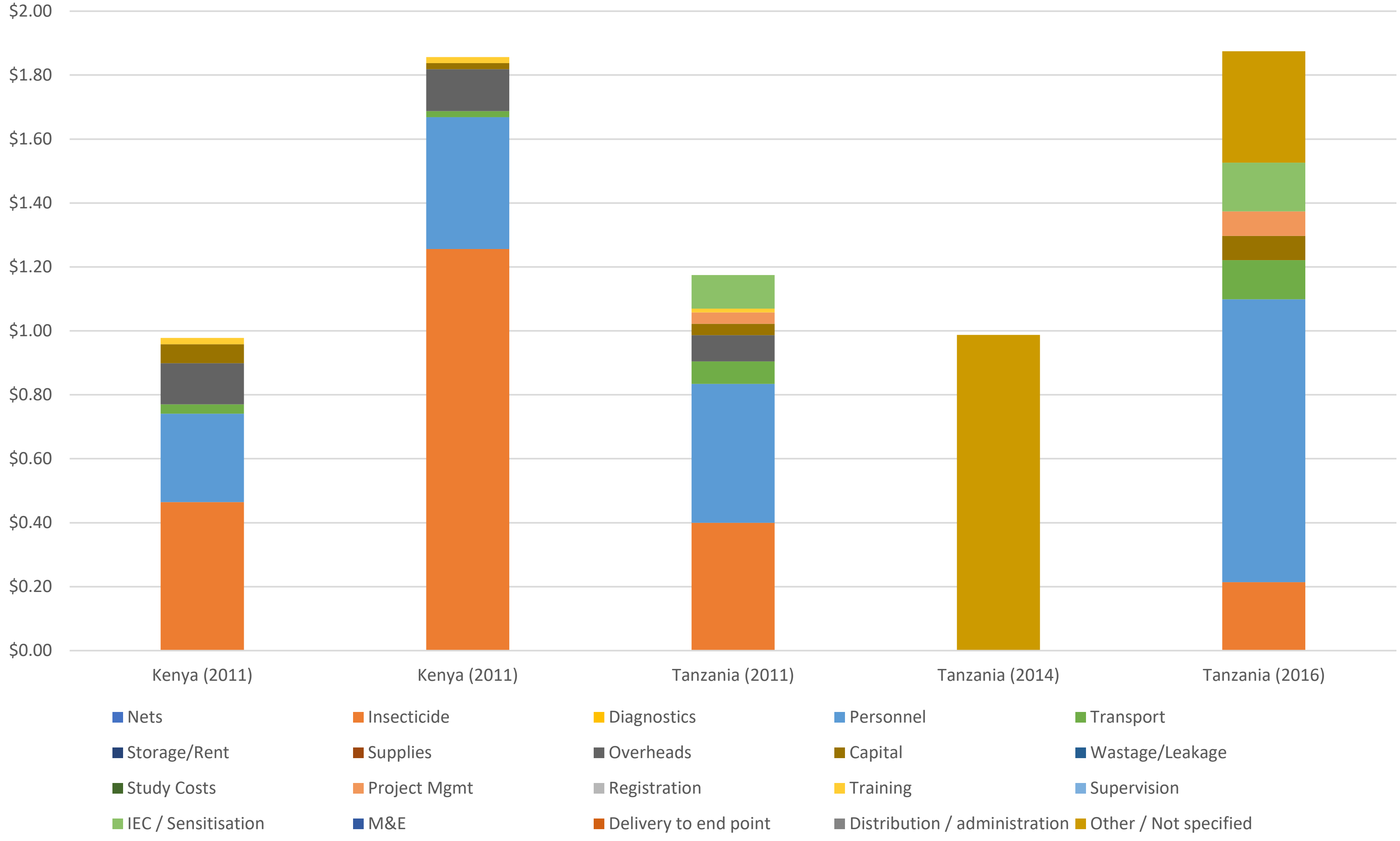

Supplement: Appendix 6 [file mmc6.pdf]
