# Supplementary material for: Costs and Cost-Effectiveness of Malaria Control Interventions: A Systematic Literature Review
Source: Value Health. 2021 Aug;24(8):1213–22. doi: 10.1016/j.jval.2021.01.013 (PMC8324482; doi:10.1016/j.jval.2021.01.013)
Supplement: Appendix 7 [file mmc7.pdf]

Appendix 7: Characteristics and results of the eligible studies considering chemoprevention in special risk groups

	Country	Setting	Delivery platform	Population group targeted (number in study)	Perspective	Cost type	Unit cost or cost-effectiveness estimate (US$ 2018)	Output or health outcome measure
Seasonal chemoprevention in children (SMC)								
Bojang et al (2011)[46]	Gambia	Rural	RCH trekking team	Children ≤ 6 years old (6076)	Provider	Financial	3.47	per first dose at all 3 treatment rounds
			RCH trekking team	Children ≤ 6 years old (6076)	Provider	Economic	4.06	per first dose at all 3 treatment rounds
			Community by VHW	Children ≤ 6 years old (6076)	Provider	Financial	1.44	per first dose at all 3 treatment rounds
			Community by VHW	Children ≤ 6 years old (6076)	Provider	Economic	1.91	per first dose at all 3 treatment rounds
Conteh et al (2010)[49]	Ghana	Mixed	Community by CHV	Children 3-59 months (2451)	Provider	Economic	9.58-17.30	per first dose at all rounds (3 or 6 rounds depending on antimalarial drug regimen)
					Provider	Economic	79.29-247.81	per malaria clinical case averted (intervention cost only)
					Provider	Economic	75.97-244.17	per malaria clinical case averted (net of treatment cost savings)
					Societal	Economic	71.37-239.56	per malaria clinical case averted (net of treatment cost savings)
Nonvignon et al (2016)[48]	Ghana	Mixed	Households by CHV	Children 3-59 months (118,208)	NR	Financial	10.24	per person receiving all 4 rounds
					Provider	Economic	23.88	per person receiving all 4 rounds
					Societal	Economic	71.39	per person receiving all 4 rounds
					Provider	Economic	113.48	per malaria episode averted
					Societal	Economic	339.16	per malaria episode averted
					Provider	Economic	3496.26	per death averted
					Societal	Economic	10449.50	per death averted
Patouillard et al (2011)[47]	Ghana	Rural	Outpatient clinic	Children 3-59 months (964)	Provider	Economic	5.77	per first dose at all 4 treatment rounds
			EPI	Children 3-59 months (964)	Provider	Economic	6.61	per first dose at all 4 treatment rounds
			Households by VHW	Children 3-59 months (964)	Provider	Economic	5.36	per first dose at all 4 treatment rounds
			Outpatient & EPI	Children 3-59 months (964)	Provider	Economic	6.17	per first dose at all 4 treatment rounds
Pitt et al (2017)[50]	Senegal	Mixed	Households by CHV	Children 3 months to 10 years (180,000)	Provider	Financial	0.47-0.53	per monthly round
					Provider	Financial	1.40-1.60	per person receiving all 3 rounds
					Provider	Economic	0.58-0.63	per monthly round

					Provider	Economic	1.74-1.89	per person receiving all 3 rounds
Intermittent preventive treatment in pregnant women (IPTp)								
Fernandes et al (2016)[57]	Burkina Faso, Ghana, Mali, Gambia	Mixed	ANC	Pregnant women (5354)	Provider	Economic	0.86	per dose
					Provider	Economic	(-193.04) - (-190.89)	per DALY averted by IPTp-AQ compared to delivery of IPTp-SP
Mbonye et al (2008)[52]	Uganda	Urban	Health centre, community	Pregnant women (2785)	Societal	Economic	3.02-3.31	per full regimen across delivery platforms
					Societal	Economic	1.38	per DALY averted by community delivery compared to health centre delivery
Orobaton et al (2016)[53]	Nigeria	Rural	Households by CHV	Pregnant women (9427)	Provider	NR	5.98-9.97	per person protected
					Provider	NR	1.14-1.80	per dose
Sicuri et al (2010)[54]	Mozambique	Rural	ANC	Pregnant women (1000)	Societal	Economic	50.17	per DALY averted
Intermittent preventive treatment in infants (IPTi)								
Conteh et al (2010)[55]	Ghana, Kenya, Mozambique, Tanzania	Mixed	EPI	Infants 3-15 months (multiple trial sizes)	Provider	Economic	0. 16-1.80	per dose delivered
					Provider	Economic	0.86- 22.46	per malaria episode averted[1]
					Provider	Economic	3.51- 47.95	per DALY averted
Hutton et al (2009)[56]	Mozambique, Tanzania	Urban	EPI	Infants <9 months (36,000 & 55,000)	Provider	Financial	0.10-0.13	per dose
					Provider	Economic	0.16-0.19	per dose
					Provider	Economic	4.63-14.00	per DALY averted
					Provider	Economic	2.00-5.88	per malaria episode averted
					Provider	Economic	125.30-376.40	per death averted
Manzi et al (2008)[51]	Tanzania	Urban	EPI	Infants 2-9 months (NR)	Provider	Financial	0.23	per dose
					Provider	Economic	0.29	per dose

Note: EPI: Expanded Programme on Immunization; NR: not reported by the study; ANC: antenatal care clinic; DALY: disability adjusted life year; Mixed refers to rural and urban study settings; CHV: community health volunteers; RCH: reproductive and child health.

[1] based on the trials that were efficacious
