# Supplementary material for: Costs and Cost-Effectiveness of Malaria Control Interventions: A Systematic Literature Review
Source: Value Health. 2021 Aug;24(8):1213–22. doi: 10.1016/j.jval.2021.01.013 (PMC8324482; doi:10.1016/j.jval.2021.01.013)
Supplement: Appendix 8 [file mmc8.pdf]

Appendix 8

A) . Breakdown of economic cost per person protected with full course+ of chemoprevention, by cost centre and study country focus (constant 2018 US$) When no breakdown available, unit cost data represented using a single colour

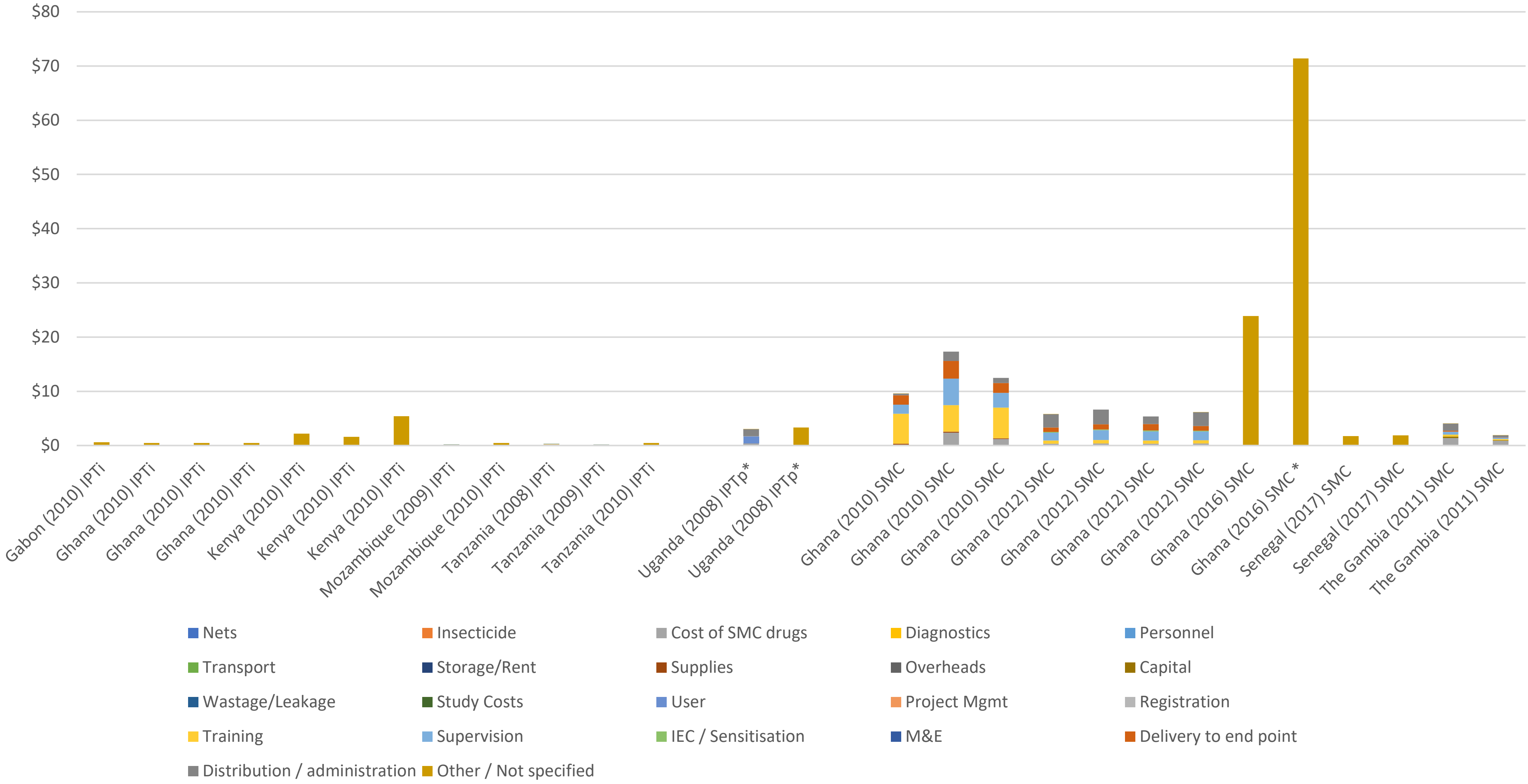

B) Breakdown of economic cost per person protected with full course⁺ of chemoprevention, by cost centre and study country focus (constant 2018 US$) When no breakdown available, unit cost data represented using a single colour (excluding Nonvignon et al, Ghana SMC 2016)

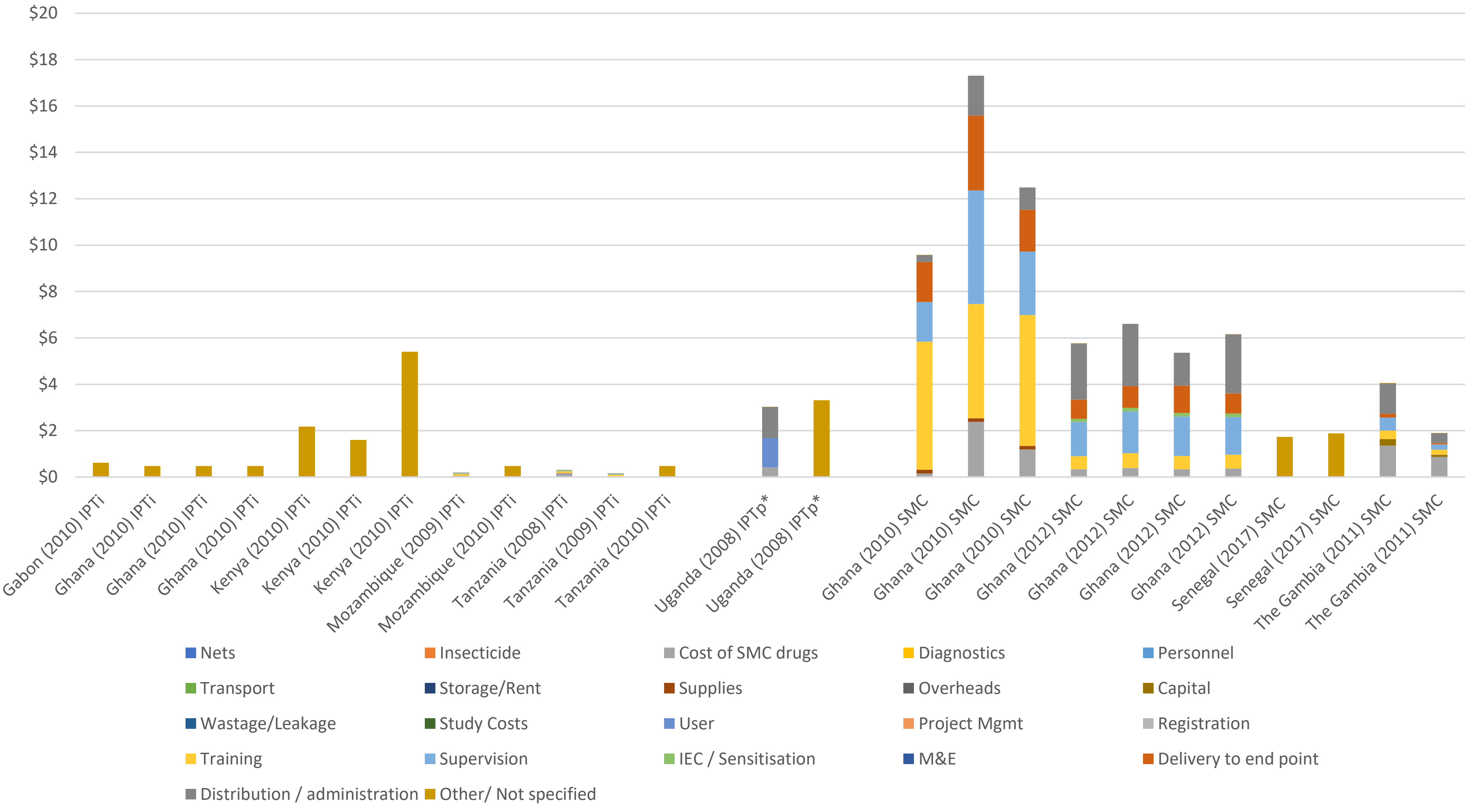

⁺Full course – mix of cost per 1st dose of all 3 treatments / per fully adherent child / per child treated per year / per full regimen. For IPTi it was per dose * 3. * Societal costs (default is provider only costs)
