# Supplementary material for: Costs and Cost-Effectiveness of Malaria Control Interventions: A Systematic Literature Review
Source: Value Health. 2021 Aug;24(8):1213–22. doi: 10.1016/j.jval.2021.01.013 (PMC8324482; doi:10.1016/j.jval.2021.01.013)
Supplement: Appendix 9 [file mmc9.pdf]

Appendix 9: Characteristics and results of the eligible studies considering Diagnostics and Treatment strategies

	Country	Setting	Intervention	Delivery platform	Population group targeted (number in study)	Perspective	Cost type	Unit cost or CE estimate (US$ 2018)	Output or health outcome measure
**Diagnostics**									
Ansah et al (2013)[58]	Ghana	Rural	RDT, microscopy	Public health facility with microscopy services; public health facility using presumptive diagnosis only.	Presumptive cases (7263)	Societal	Economic	26.10-28.30	per case diagnosed
						Provider	Economic	18.00-20.10	per case diagnosed and treated
						Societal	Economic	26.10-28.30	per case diagnosed and treated
						Provider	Economic	18.00	per additional case diagnosed and correctly treated compared to presumptive diagnosis
						Societal	Economic	14.30	per additional case diagnosed and correctly treated compared to presumptive diagnosis
						Provider	Economic	7.80	per additional case diagnosed and correctly treated compared to microscopy diagnosis
						Societal	Economic	6.20	per additional case diagnosed and correctly treated compared to microscopy diagnosis
Batwala et al (2011)[59]	Uganda	Rural	RDT, Microscopy	Public health center	All fever cases in high or low transmission settings (1627)	Provider	Economic	1.44	per case diagnosed with RDT
						Provider	Economic	1.71	per case diagnosed with microscopy
						Societal	Economic	5.64	per case diagnosed and treated with RDT
						Societal	Economic	6.27	per case diagnosed and treated with microscopy
						Societal	Economic	1.31	per additional case diagnosed and treated with RDT compared to presumptive diagnosis across transmission settings
						Societal	Economic	1.66	per additional case diagnosed and treated with microscope compared to presumptive diagnosis across transmission settings
						Societal	Economic	4.90-6.60	per additional case diagnosed and correctly treated with RDT compared to presumptive diagnosis across transmission settings
						Societal	Economic	8.60-14.50	per additional case diagnosed and correctly treated with microscope compared to presumptive diagnosis across transmission settings
Chanda et al (2009)[60]	Zambia	Mixed	RDT	Public health facility	Presumptive (6685)	Provider	Economic	6.10	per case diagnosed with RDT
			Microscopy	Public health facility	Presumptive cases (10460)	Provider	Economic	10.60	per case diagnosed with microscopy
			RDT	Public health facility	Presumptive (6685)	Provider	Economic	3.50	per additional case correctly diagnosed with RDT compared to presumptive diagnosis
			Microscopy	Public health facility	Presumptive cases (10460)	Provider	Economic	12.40	per additional case correctly diagnosed with microscopy compared to presumptive diagnosis
de Oliveira et al (2010)[61]	Brazil	Rural	RDT, Microscopy	Public health center and mobile clinic	Hypothetical cohort of all	Provider	Economic	6.40	per case diagnosed with RDT

					individuals with fever (33491)				
						Provider	Economic	8.50	per case diagnosed with microscopy
						Provider	Economic	2.10	per additional case diagnosed with microscopy compared to RDT
						Provider	Economic	687.40	per additional adequately diagnosed case with microscopy compared to RDT
de Oliveira et al (2012)[62]	Brazil	NR	RDT, Microscopy	NR - public sector	Hypothetical cohort of all individuals with fever (2702)	Provider	Financial	13.30-17.30	per case diagnosed with RDT
								38.80	per case diagnosed with microscopy
								8.20	per additional adequately diagnosed case using shared microscopy compared to RDT
Faye (2010)[63]	Senegal	NR	RDT	Public health center and post	Presumptive cases tested and treated with ACT (379)	Provider	Financial	0.60	per case tested
Hansen et al (2015)[65]	Afghanistan	Rural	RDT	NGO health facility with and without microscopy services	Presumptive cases in low or moderate transmission settings (5749)	Provider	Economic	2.25-9.73	per case diagnosed across facility type and transmission settings
						Societal	Economic	11.12-15.80	per case diagnosed and appropriately treated across facility type and transmission settings
						Provider	Economic	2.93	per additional case diagnosed with RDT and appropriately treated compared to presumptive diagnosis
						Societal	Economic	5.27	per additional case diagnosed with RDT and appropriately treated compared to presumptive diagnosis
Hansen et al (2017a)[64]	Uganda	Rural	RDT	Private drug shops selling subsidized ACT	Presumptive malaria cases (506)	Provider	Economic	3.51	per case diagnosed and treated
						Societal	Economic	10.55	per case diagnosed and treated
						Provider	Economic	0.62	per additional case appropriately treated following introduction of RDT compared to presumptive treatment
						Societal	Economic	4.29	per additional case appropriately treated following introduction of RDT compared to presumptive treatment
Hansen et al (2017b)[66]	Uganda		RDT	Public CHW	Presumptive cases in meso endemic moderate to high transmission or low transmission settings (1000)	Provider	Economic	6.35-25.73	per case diagnosed with RDT and treated across settings
						Societal	Economic	9.80-31.30	per case diagnosed with RDT and treated across settings
						Provider	Economic	3.40-14.90	per additional case diagnosed and appropriately treated compared to presumptive treatment across settings

						Societal	Economic	4.00-16.70	per additional case diagnosed and appropriately treated compared to presumptive treatment across settings
Lemma et al (2011)[67]	Ethiopia	Urban	RDT	Public health post CHW	Presumptive cases (2422)	Provider	Financial	2.04	per correctly diagnosed and treated case
						Provider	Financial	0.71	per additional correctly diagnosed and treated case with *Plasmodium falciparum* + *Plasmodium vivax* RDT compared to *Plasmodium falciparum*-only RDT
Lubell et al (2007)[68]	United Republic of Tanzania (Mainland)	Rural	RDT	Public hospital	Presumptive cases in low or high transmission settings (2416)	Provider	Economic	9.00-32.50	per additional case diagnosed and correctly treated with RDT compared to microscopy
Matangila et al (2014)[69]	Democratic Republic of the Congo	mixed	RDT	NGO hospital	Pregnant women at first antenatal care visit (333)	Provider	Economic	1.26	per case diagnosed
			Microscopy	NGO hospital	Pregnant women at first antenatal care visit (333)	Provider	Economic	2.86	per case diagnosed
						Provider	Economic	1.59	per case correctly diagnosed with microscopy compared to RDT
						Provider	Economic	69.18	per additional case correctly diagnosed with microscopy compared to RDT
Ogunniyi et al (2016)[70]	Nigeria	Urban	RDT	Public health facility	Presumptive malaria cases (502)	NR	Economic	6.24	per test
			Microscopy	Public health facility	Presumptive malaria cases (502)	NR	Economic	2.16-11.42	per test
Parikh et al (2010)[75]	Nigeria	Peri-urban	Microscopy	Faith-based hospital	Presumptive malaria cases (304)	Provider	NR	4.40	per slide
Rolland et al (2006)[71]	South Sudan, Ethiopia	NR	RDT	iNGO CHW	Hypothetical fever cases in areas with different malaria prevalence (10,000)	Provider	NR	10.36	per case diagnosed
			RDT	iNGO CHW	Hypothetical fever cases in areas with different malaria prevalence (10,000)	Provider	NR	0.65	per additional false positive case averted with RDT compared to presumptive diagnosis
Tawiah et al (2016)[72]	Ghana	rural	RDT	Public health center	Children under five years with fever (11364)	Provider	Economic	11.70	per case diagnosed
					Children under five years with fever (2006)	Household	Economic	6.60	per case diagnosed
					Children under five years with fever (11364)	Provider	Economic	2.80	per additional case tested with RDT and treated compared to presumptive diagnosis

					Children under five years with fever (11364)	Societal	Economic	1.70	per additional case tested with RDT and treated compared to presumptive diagnosis
					Children under five years with fever (11364)	Provider	Economic	20.80	per additional case tested with RDT and appropriately treated compared to presumptive diagnosis
					Children under five years with fever (11364)	Societal	Economic	12.30	per additional case tested with RDT and appropriately treated compared to presumptive diagnosis
Willcox et al (2009)[73]	Mali	Rural	RDT	CHW public	Presumptive malaria cases (301)	NR	NR	2.45	per case diagnosed and treated
Yukich et al (2010)[74]	Tanzania	Urban	RDT	Public health facility	Presumptive malaria cases (333)	Provider	Financial	0.05	per case diagnosed (excluding test kit cost)
						Household	Economic	1.19	Per case diagnosed
						Provider	Economic	4.25	per case diagnosed and treated
Treatment									
Abotsi et al (2012)[76]	Ghana	Mixed	Treatment of uncomplicated and severe cases	Public health center	Confirmed cases in infants (65703)	Provider	Economic	4.62	per uncomplicated case treated
				Public hospital	Confirmed cases in infants (NR)	Provider	Economic	89.93	per severe case treated
				Public health center	Confirmed cases in infants (NR)	Household	Financial	12.33	per uncomplicated case treated
				Public hospital	Confirmed cases in infants (NR)	Household	Financial	56.41	per severe case treated
Ayieko et al (2009)[77]	Kenya	NR	Treatment of severe cases	Public hospitals	Children under 5 years (25—44)	Provider	Economic	60.88-123.30	per case treated across study areas
			Treatment of severe cases	Faith-based hospitals	Children under 5 years (30)	Provider	Economic	54.48-113.75	per case treated across study areas
			Treatment of severe cases	Public hospitals	Children under 5 years (NR)	Societal	Economic	97.02-174.89	per case treated across study areas
			Treatment of severe cases	Faith-based hospitals	Children under 5 years (NR)	Societal	Economic	115.57	per case treated across study areas
Bôtto-Menezes et al (2016)[94]	Brazil	Urban	Treatment of uncomplicated or severe malaria	Public hospital	Pregnant and postpartum women (15)	Provider	Economic	108.22	per uncomplicated case treated
				Public hospital	Pregnant and postpartum women (15)	Provider	Economic	376.86	per severe case treated
				Public hospital	Pregnant and postpartum women (15)	Provider	Economic	125.62	per severe case treated per day
				Public health facility	Pregnant and postpartum women (65)	Household	Economic	51.43	per uncomplicated case treated
				Public health facility	Pregnant and postpartum women (8)	Household	Economic	235.76	per severe case treated
Chanda et al (2007)[79]	Zambia	NR	Treatment of uncomplicated	Public health center	All (55509)	Provider	Economic	8.00-9.50	per uncomplicated case treated across antimalarial types

			or severe cases using different antimalarials						
						Provider	Economic	12.80-26.81	per severe case treated across antimalarial types
						Provider	Economic	5.30	per additional uncomplicated case treated with AL to SP
						Provider	Economic	(-14.90)	per additional severe case treated with AL+Q compared to SP+Quinine
Chanda et al (2011)[78]	Zambia	Rural	Treatment of uncomplicated cases	CHW	All (9552)	Provider	Economic	0.27	per case appropriately treated
				Public health facility	All (29932)	Provider	Economic	1.50	per case appropriately treated
				CHW	All (9552)	Provider	Economic	4.67	per case appropriately diagnosed and treated
				Public health facility	All (29932)	Provider	Economic	6.24	per case appropriately diagnosed and treated
				CHW	All (10100)	Provider	Economic	4.89	per additional case appropriately diagnosed and treated with CHW compared to public facility
Collins et al (2014)[80]	Senegal,	Rural	Treatment of uncomplicated malaria	iCCM	Children 2–59 months (239,861)	Provider	NR	4.72	per case treated (includes medicines, supplies, management, supervision, training)
	Zambia	Rural	Treatment of uncomplicated malaria	iCCM	Children 2–59 months (78,797)	Provider	NR	4.49	per case treated (includes medicines, supplies, management, supervision, training)
	Senegal, Zambia, Malawi, Cameroon, Democratic Republic of the Congo, Sierra Leone, South Sudan	Rural	Treatment of uncomplicated malaria	iCCM	Children 2–59 months (25,114-615,149)	Provider	NR	0.44-0.98	per case treated (includes test and medicine only)
Comfort et al (2014)[81]	Zambia	Rural	Treatment for uncomplicated malaria	Faith-based hospital	All (373)	Provider	Financial	77.96	per uncomplicated case treated
		Rural	Treatment for severe malaria	Faith-based hospital	All (373)			75.70	per severe case treated
		Rural	Treatment for malaria with anaemia	Faith-based hospital	All (373)			46.36	per case with anaemia
		Urban	Treatment for malaria with anaemia	Public hospital	All (783)			72.83	per case with anaemia
		Rural	Treatment for malaria with severe anaemia	Faith-based hospital	All (373)			100.40	per case with severe anaemia
		Urban	Treatment for malaria with severe anaemia	Public hospital	All (783)			74.19	per case with severe anaemia

		Rural	Treatment for cerebral malaria	Faith-based hospital	All (373)			48.95	per cerebral case
		Urban	Treatment for cerebral malaria	Public hospital	All (783)			74.19	per cerebral case
		Rural	Treatment for cerebral malaria with moderate anaemia	Faith-based hospital	All (373)			136.96	per cerebral case with moderate anaemia
		Urban	Treatment for cerebral malaria with moderate anaemia	Public hospital	All (783)			75.70	per cerebral case with moderate anaemia
Daviaud et al (2017)[82]	Ethiopia	Mixed	Treatment of uncomplicated malaria	iCCM	Children 2 to less than 5 years (NR)	Provider	Economic	14.10	per case treated
	Ghana	Mixed	Treatment of uncomplicated malaria	iCCM	Children 2 to less than 5 years (NR)	Provider	Economic	14.95	per case treated
	Mali,	Mixed	Treatment of uncomplicated malaria	iCCM	Children 2 to less than 5 years (NR)	Provider	Economic	9.75	per case treated
	Malawi	Mixed	Treatment of uncomplicated malaria	iCCM	Children 2 to less than 5 years (NR)	Provider	Economic	3.18	per case treated
	Mozambique	Mixed	Treatment of uncomplicated malaria	iCCM	Children 2 to less than 5 years (NR)	Provider	Economic	6.68	per case treated
	Niger	Mixed	Treatment of uncomplicated malaria	iCCM	Children 2 to less than 5 years (NR)	Provider	Economic	6.04	per case treated
Davis et al (2011)[95]	Papua New Guinea	Rural	Treatment of uncomplicated *Pf* and *Pv* cases using different AM	Clinics	Children 6 months to 5 years (33-104)	Societal	Financial	4.30-6.10	per uncomplicated case treated across different antimalarials
								(-0.21)-11.90	per additional uncomplicated case successfully treated using ACT compared to CQ+SP
								67.90-87.30	per life year saved using AL compared to other antimalarials
Escribano et al (2017)[83]	Ghana	Rural Mixed	Treatment of uncomplicated malaria	Community (iCCM or CHPS)	Children under 5 years with fever, diarrhoea or cough (NR)	Societal	Economic	5.30-10.10	per case diagnosed and treated across settings
						Provider	Economic	5.17-9.90	per case diagnosed and treated across settings
						Household	Economic	0.04-1.92	per case diagnosed and treated across settings
Ezenduka et al (2017)[84]	Nigeria	Urban	Treatment of uncomplicated cases	Hospital	Uncomplicated cases (NR)	Provider	Economic	34.01	per case treated (no comedication)
								38.50	per case treated (with comedication)

Ferrari et al (2015)[85]	Republic Democratic du Congo	Urban, rural, mixed	Treatment for severe malaria using different AM	Faith based, Private, Public, Health centers, Hospitals	Cases above two months of age (350-399)	Provider	Financial	4.74-65.27	per case treated across different antimalarials and settings
Kyaw et al (2014)[86]	Thailand	Rural	Treatment of *Plasmodium falciparum* uncomplicated malaria	Public hospital	Malaria inpatient cases (100)	NR	Financial	1.00-1.15	per case treated across different antimalarials
			Treatment of *Plasmodium falciparum* severe malaria					3.30-3.50	per case treated across different antimalarials
Maka et al (2016)[87]	Cameroon	rural	Treatment of severe malaria using different AM and dosage	Public hospital	Children aged 3 months to 15 years (30)	Provider	Financial	68.40-69.70	per case treated across different antimalarials
								29.60-37.80	per additional measure of parasite reduction in 24 hours using different antimalarials compared to quinine
Moore et al (2017)[96]	Papua New Guinea	NR	Treatment of uncomplicated *Plasmodium falciparum*	Public health center	Children (186-267)	Societal	Financial	8.30-18.80	per case treated across different antimalarials
			Treatment of uncomplicated *Plasmodium vivax*					8.10-19.40	per case treated across different antimalarials
			Treatment of uncomplicated *Plasmodium falciparum*					10.50	per additional case treated with artemisinin-naphthoquine compared to artemether-lumefantrine
			Treatment of uncomplicated *Plasmodium vivax*					11.30	per additional case treated with artemisinin-naphthoquine compared to artemether-lumefantrine
			Treatment of uncomplicated *Plasmodium falciparum*					476.10	per year of life saved with artemisinin-naphthoquine compared to artemether-lumefantrine
			Treatment of uncomplicated *Plasmodium vivax*					16.20	per year of life saved with artemisinin-naphthoquine compared to artemether-lumefantrine
Mori et al (2014)[88]	Republic of Tanzania (Mainland)	Urban	Treatment of uncomplicated malaria	Public hospital	Children aged 0.5-4.9 years (1263-7076)	Provider	Economic	9.20-9.30	per case treated across different antimalarials
			Treatment of severe malaria	Public hospital	Children aged 0.5-4.9 years (1263-7076)	Provider	Economic	91.40	per case treated
			Treatment of uncomplicated malaria	Public hospital	Children aged 0.5-4.9 years (1263-7076)	Provider	Economic	13.50	per DALY averted using dihydroartemisin-piperaquine compared to do nothing under a highly patient adherence scenario

Nonvignon et al (2012)[89]	Ghana	rural	Treatment of uncomplicated malaria	Public CHW	Children aged 0.2-4.9 years (12000)	Societal	Economic	3025.10	per death averted compared to business as usual (no CHW)
								106.00	per DALY averted compared to business as usual (no CHW)
Onwujekwe et al (2007)[91]	Nigeria	rural	Treatment of uncomplicated malaria	Public CHW	Presumptive cases (293-392)	Provider	Financial	1.10-1.40	per villager across ages and settings
						Provider	Financial	13.80-17.50	per patient treated across ages and settings
						Provider	Economic	1.20-1.60	per villager across settings
						Household	Financial	0.01-0.06	per villager across settings
						Household	Economic	0.60-0.70	per villager across settings
						Societal	Economic	1.20-1.60	per villager across settings
Onwujekwe et al (2013)[90]	Nigeria	urban	Treatment of uncomplicated and severe cases	Public hospital and health centres, mission hospital	Children less than five years diagnosed and treated for malaria (500)	Societal	Economic	190.10	per uncomplicated case
						Societal	Economic	2082.60	per severe case
						Provider	Economic	32.80	per uncomplicated case
						Provider	Economic	51.90	per severe case
						Household	Economic	13.60	per uncomplicated case
						Household	Economic	25.10	per severe case
Wiseman et al (2006)[92]	Republic of Tanzania (mainland)	mixed	Treatment of uncomplicated cases	Public hospital	Children aged less than 5 years (1811)	Societal	Economic	13.71-29.08	per case diagnosed and treated across different antimalarials
						Provider	Economic	7.13-8.34	per case diagnosed and treated across different antimalarials
						Household	Economic	5.36-21.90	per case diagnosed and treated across different antimalarials
						Societal	Economic	(-31.42) -(-27.59)	per additional malaria episode averted using ACT compared to monotherapy
						Provider	Economic	(-0.29)-6.88	per additional malaria episode averted using ACT compared to monotherapy
Yeung et al (2008)[93]	Cambodia	rural	Treatment for uncomplicated *Plasmodium falciparum*	Community (public mobile clinics)	Malaria cases (NR)	Provider	Economic	0.80	per person
				Community (CHW)				1.80	per person
				Community (public mobile clinics)				4.70	per case tested
				Community (CHW)				3.10	per case tested
				Community (public mobile clinics)				22.60	per case treated
				Community (CHW)				13.20	per case treated

RDT: rapid diagnostic test; CHW: community health workers; iNGO: international non-governmental organization; CE: cost-effectiveness; NR: not reported; iCCM: integrated community case management; CHPS: community-based health planning and services; DALY: disability adjusted life year; AL+Q: artemether-lumefrantine+Quinine; CQ+SP: chloroquine+sulphadoxine-pyrimethamine; ACT: artemisinin-combination therapy
