# Supplementary material for: Costs and Cost-Effectiveness of Malaria Control Interventions: A Systematic Literature Review
Source: Value Health. 2021 Aug;24(8):1213–22. doi: 10.1016/j.jval.2021.01.013 (PMC8324482; doi:10.1016/j.jval.2021.01.013)
Supplement: Appendix 10 [file mmc10.pdf]

Appendix 10: Breakdown of economic cost per case diagnosed, by cost centre and study country focus (constant 2018 US$)
When no breakdown available, unit cost data represented using a single colour

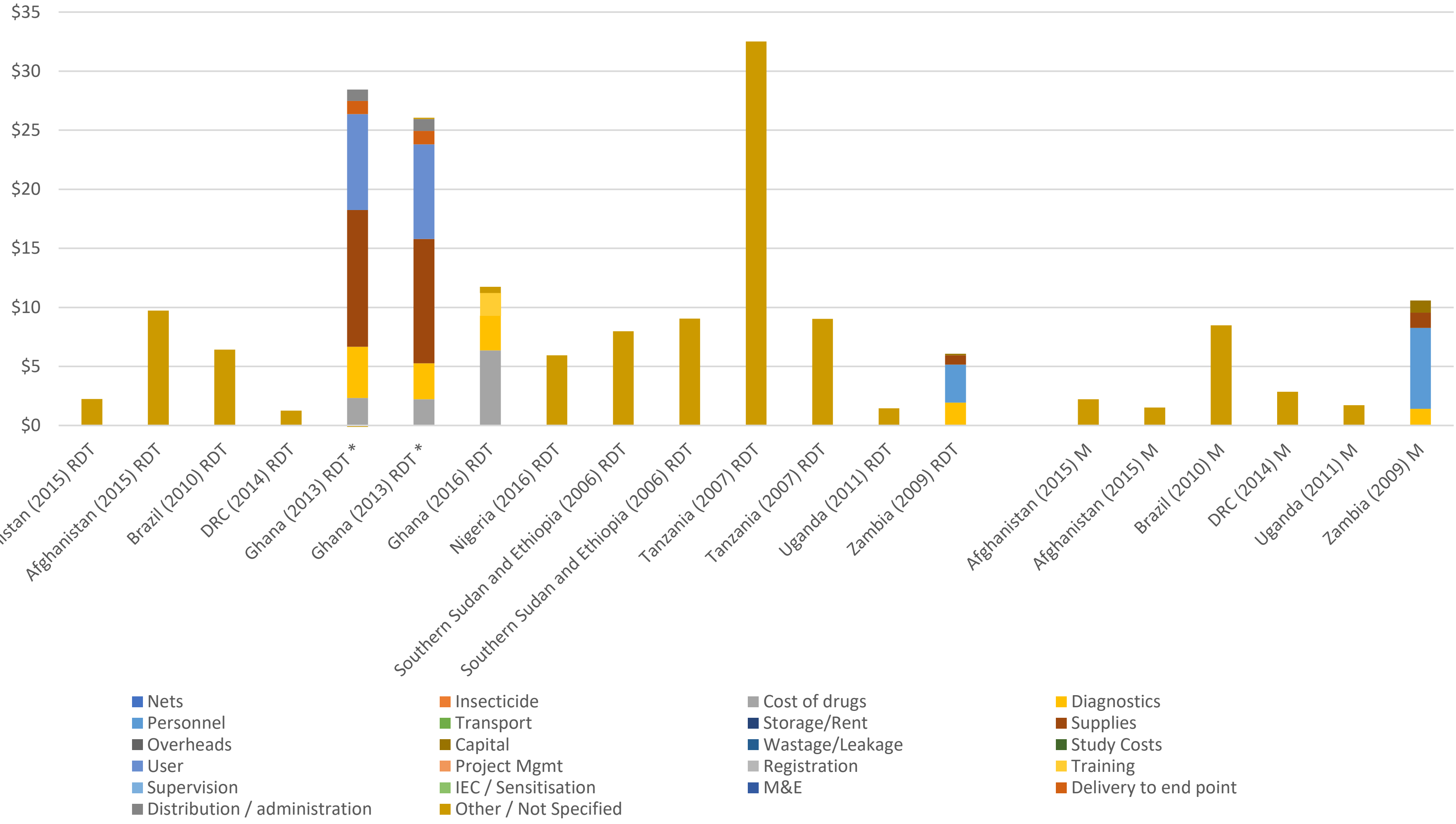

RDT- rapid diagnostic test, M – Microscopy,

* Societal costs (default is provider only costs)
