# Supplementary material for: Costs and Cost-Effectiveness of Malaria Control Interventions: A Systematic Literature Review
Source: Value Health. 2021 Aug;24(8):1213–22. doi: 10.1016/j.jval.2021.01.013 (PMC8324482; doi:10.1016/j.jval.2021.01.013)
Supplement: Appendix 11 [file mmc11.pdf]

Appendix 11

A) Breakdown of economic cost per uncomplicated malaria episode treated, by cost centre and study country focus (constant 2018 US$). When no breakdown available, unit cost data represented using a single colour

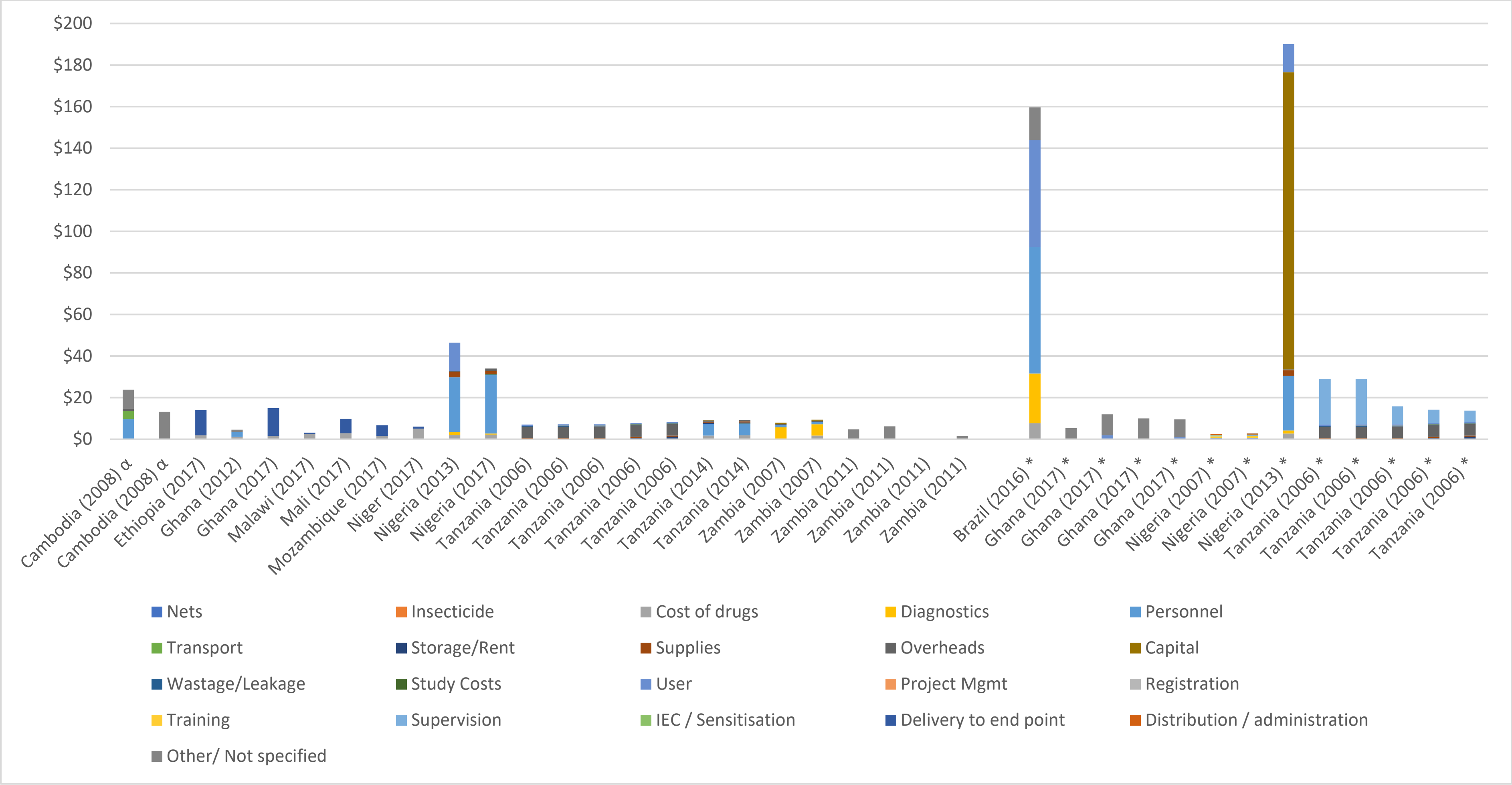

* societal perspective (default is provider)

α *Plasmodium vivax* (default is *Plasmodium falciparum*)

B) Breakdown of economic cost per severe malaria episode treated, by cost centre and study country focus (constant 2018 US$) minus outlier Onwujekwe, Nigeria 2013). When no breakdown available, unit cost data represented using a single colour

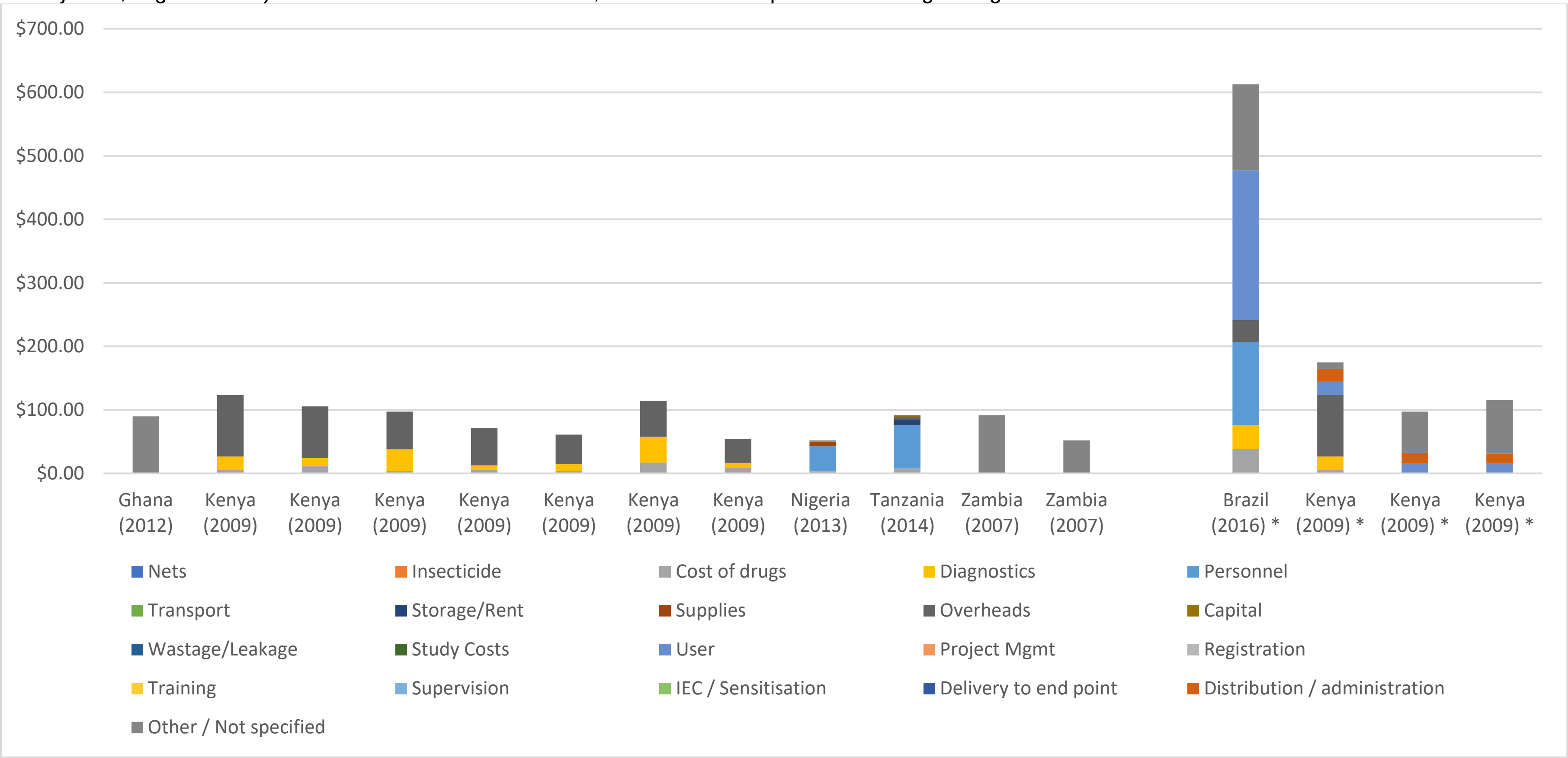

* societal perspective (default is provider)
