# Supplementary material for: Costs and Cost-Effectiveness of Malaria Control Interventions: A Systematic Literature Review
Source: Value Health. 2021 Aug;24(8):1213–22. doi: 10.1016/j.jval.2021.01.013 (PMC8324482; doi:10.1016/j.jval.2021.01.013)
Supplement: Appendix 12 [file mmc12.pdf]

Appendix 12: Characteristics and results of the eligible studies considering surveillance interventions.

	Country	Setting	Intervention	Delivery platform	Population group targeted (number in study)	Perspective	Cost type	Unit cost or CE estimate (US$ 2018)	Output or health outcome measure
Surveillance systems for epidemics									
Mueller et al (2009)[103]	Kenya and Uganda	Rural	Early detection system of epidemics	Representative sentinel sites reported daily tallies of malaria cases on a weekly basis to District Health Management Teams	20 sentinel health facilities (five health centres in each of the four pilot districts)	Provider	Economic	0.04- 0.05	per person per year across settings
						Provider	Economic	124.38-623.75	per DALY averted across settings
Worrall et al (2008)[104]	Zimbabwe	Mixed	IRS informed by an Integrated Malaria Early Warning and Response System	Incorporating climate information into the decision-making process of malaria control programmes	Households (24,236)	Provider	Economic	1.47	per person protected
								1.28-3.29	per case averted in high transmission years
								157.68-389.82	per case averted in low transmission years
Entomological surveillance									
Chaki et al (2012)[105]	Tanzania	Urban	Mosquito trapping schemes	Community-based surveillance scheme using Ifakara Tent Trap (CB-ITT)	Inhabitants (2.65 million)	Provider	NR	5.15	per night of sampling
				Community-based surveillance scheme using Ifakara Tent Trap (CB-ITT)	Inhabitants (2.65 million)	Provider	NR	129.12	per specimen of An. Gambiae s.l.
				Quality assurance surveillance teams conducting entomological surveys using human landing catches (QA-HLC)	Inhabitants (2.65 million)	Provider	NR	108.99	per night of sampling
				Quality assurance surveillance teams conducting entomological surveys using human landing catches (QA-HLC)	Inhabitants (2.65 million)	Provider	NR	216.05	per specimen of An. Gambiae s.l.
				Quality assurance surveillance teams conducting entomological surveys using ITT (QA-ITT)	Inhabitants (2.65 million)	Provider	NR	60.57	per night of sampling
				Quality assurance surveillance teams conducting entomological surveys using ITT (QA-ITT)	Inhabitants (2.65 million)	Provider	NR	659.08	per specimen of An. Gambiae s.l.
Sikaala et al (2014)[106]	Zambia	Rural	Mosquito trapping schemes	Community-based surveillance scheme using light traps (LT) (CB-LT)	Households (14,000)	Provider	NR	14.82	per person-night of sampling
				Community-based surveillance scheme using light traps (LT) (CB-LT)	Households (14,000)	Provider	NR	5.78	per specimen of An. Funestus caught
				Community-based surveillance scheme using Ifakara Tent Traps (CB-ITT)	Households (14,000)	Provider	NR	19.62	per person-night of sampling

				Community-based surveillance scheme using Ifakara Tent Traps (CB-ITT)	Households (14,000)	Provider	NR	30.52	per specimen of An. Funestus caught
				Quality assurance surveillance teams conducting entomological surveys using LT (QA-LT)	Households (14,000)	Provider	NR	315.45	per person-night of sampling
				Quality assurance surveillance teams conducting entomological surveys using LT (QA-LT)	Households (14,000)	Provider	NR	153.91	per specimen of An. Funestus caught
				Quality assurance surveillance teams conducting entomological surveys using ITT (QA-ITT)	Households (14,000)	Provider	NR	293.32	per person-night of sampling
				Quality assurance surveillance teams conducting entomological surveys using ITT (QA-ITT)	Households (14,000)	Provider	NR	183.34	per specimen of An. Funestus caught
				Quality assurance surveillance teams conducting entomological surveys using human landing catches (QA-HLC)	Households (14,000)	Provider	NR	150.75	per person-night of sampling
				Quality assurance surveillance teams conducting entomological surveys using human landing catches (QA-HLC)	Households (14,000)	Provider	NR	11.45	per specimen of An. Funestus caught
Active case detection									
Drake et al (2011)[98]	Kenya	Mixed	Proactive case detection	Primary schools	Children in 101 primary schools (3685)	Provider	Financial	7.60	per person tested and if positive, treated (screened)
					Children in 101 primary schools (3685)	Provider	Economic	7.18	per person tested and if positive, treated (screened)
Hamainza et al (2014)[99]	Zambia	Rural	Proactive case detection	CHW	Households (14,000)	Provider	Financial	11.64	per person tested and if positive treated
								6.81	per person tested only
								8.82	per case tested and treated
Larson et al (2016)[100]	Zambia	Urban	Reactive case detection	CHW	People residing with or in surrounding of a passively detected case living in one of the studied 164 health facility catchment areas (NR)	Provider	Economic	1.25	per person covered per year
						Provider	Economic	7.48	per structure visited
						Provider	Economic	38.63	per person tested

						Provider	Economic	32.07	per case treated
Silumbe et al (2015)[101]	Zambia	Mixed	Proactive case detection	CHW	People residing in 18 health facility catchment areas (135,649)	Provider	Economic	4.79	per test administered
						Provider	Economic	37.87	per treatment administered
						Provider	Economic	9.52	per person targeted
						Provider	Economic	79.25	per malaria episode averted
						Provider	Economic	39628.04	per death averted
						Provider	Economic	974.46	per DALY averted
Zelman et al (2018)[102]	Indonesia	Rural	Reactive case detection	Microscopy-confirmed malaria case diagnosed at health facility, then surveillance staff visit village of index case	People residing within 500 meters of a case passively detected in one of the four studied sub-district health facility sites (1495)	Provider	NR	0.45	per person covered per year
								1248.68	per case detection event
								29.68	per person tested with microscopy and/or LAMP
								7494.20	per case identified with microscopy and/or LAMP
								6261.42	per additional infection detected through LAMP compared to microscopy

Note: CE: cost-effectiveness; LAMP: loop-mediated isothermal amplification; DALY: disability adjusted life year
