# Supplementary material for: Costs and Cost-Effectiveness of Malaria Control Interventions: A Systematic Literature Review
Source: Value Health. 2021 Aug;24(8):1213–22. doi: 10.1016/j.jval.2021.01.013 (PMC8324482; doi:10.1016/j.jval.2021.01.013)
Supplement: Appendix 13 [file mmc13.pdf]

Appendix 13: Characteristics of the eligible studies considering combination strategies

	Country	Setting	Intervention	Delivery platform and ownership	Target population (number in study)	Perspective	Cost type	Cost or CE estimate (US$ 2018)	Output or health outcome measure
Combinations of interventions									
Giron et al (2006)[107]	Colombia	Urban	National programme*	Public health facility, Community, Home	Presumptive cases (1970)	Societal	Economic	3.75	per person
			National programme*	Public health facility, Community, Home	Presumptive cases (1970)	Provider	Economic	450.30	per malaria case averted
			National programme* and education strategy based on social mobilization	Public health facility, Community, Home	Presumptive cases (1970)	Societal	Economic	5.79	per person
			National programme* and education strategy based on social mobilization	Public health facility, Community, Home	Presumptive cases (1970)	Provider	Economic	141.99	per malaria case averted
Hailu et al (2018)[108]	Ethiopia	Rural	LLIN	Households	Households (1338)	Provider	Economic	1.12	per person-year protected
			LLIN	Households	Households (1338)	Provider	Economic	7.40	per child under five years protected
			LLIN	Households	Households (1338)	Provider	Economic	83.39	per pregnant woman protected
			LLIN	Households	Households (1338)	Provider	Economic	5.82	per household covered
			LLIN	Households	Households (1338)	Provider	Economic	-4799.7	per additional DALY averted compared to. routine (no LLIN/IRS, trial-based CEA)
			LLIN	Households	Households (1338)	Provider	Economic	219.42	per additional DALY averted compared to. routine (no LLIN/IRS, literature-based CEA)
			IRS	Households	Households (1527)	Provider	Economic	3.25	per person-year protected
			IRS	Households	Households (1527)	Provider	Economic	21.33	per child under five years protected
			IRS	Households	Households (1527)	Provider	Economic	241.33	per pregnant woman protected
			IRS	Households	Households (1527)	Provider	Economic	16.49	per household covered
			IRS	Households	Households (1527)	Provider	Economic	-10186.6	per additional DALY averted compared to routine (no LLIN/IRS, trial-based CEA)
			IRS	Households	Households (1527)	Provider	Economic	-1507.32	per additional DALY averted compared to routine (no LLIN/IRS, literature-based CEA)
			LLINS&IRS	Households	Households (1618)	Provider	Economic	4.28	per person-year protected
			LLINS&IRS	Households	Households (1618)	Provider	Economic	28.10	per child under five years protected
			LLINS&IRS	Households	Households (1618)	Provider	Economic	316.66	per pregnant woman protected
			LLINS&IRS	Households	Households (1618)	Provider	Economic	21.74	per household covered
			LLINS&IRS	Households	Households (1618)	Provider	Economic	-14358.76	per additional DALY averted compared to. routine (no LLIN/IRS, trial-based CEA)
			LLINS&IRS	Households	Households (1618)	Provider	Economic	1487.18	per additional DALY averted compared to routine (no LLIN/IRS, literature-based CEA)
Hansen et al (2012)[109]	Uganda	Urban	IPTp	Public health facilities	Pregnant women (1604)	Provider	Financial	0.79	per person
			ITN	Public health facilities	Pregnant women (1604)	Provider	Financial	1.71	per person
			IPTp & ITN	Public health facilities	Pregnant women (1604)	Provider	Financial	2.48	per person
			ITN	Public health facilities	Pregnant women (1604)	Provider	Financial	69.66	per additional DALY averted compared to IPTp only

			IPTp & ITN	Public health facilities	Pregnant women (1604)	Provider	Financial	-68.37	per additional DALY averted compared to IPTp only
Haque et al (2014)[110]	Bangladesh	Mixed	ITN (LLIN or retreatment for ITN), diagnosis, treatment, education, surveillance system; partnerships	Multiple	Households (14 million)	Provider	Financial	0.74	per person-year protected with ITN
						Provider	Financial	0.43	per case diagnosed
						Provider	Financial	0.56	per case treated
						Provider	Financial	0.05	per person per year (health education)
Howard et al (2017)[111]	Pakistan	Urban	IRS, diagnostics using microscopy, treatment	Basic health Units	Refugees (2.5 million)	Societal	Economic	0.59	per person targeted
								0.24	per person targeted with IRS only
								0.35	per person targeted with diagnosis and treatment
								117.66	per vivax case prevented compared to no IRS
								468.52	per falciparum/mixed case prevented compared to no IRS
								93.28	per case averted compared to no IRS
								335,738.04	per death averted compared to no IRS
								1071.66	per year of healthy life gained compared to no IRS
								637.06	per DALY averted compared to no IRS
Maccario et al (2017)[112]	Mali	Urban	Education: training teachers; health guidance manual development; participatory learning activities; awareness day organization	Primary schools	Children (6413)	Provider	Financial	2.13	per person covered
			Education: training teachers; health guidance manual development; participatory learning activities; awareness day organization		Children (6413)	Provider	Economic	1.25	per person covered
			LLIN only		Children (6413)	Provider	Financial	5.53	per person covered (assumes 2 ITN per child)
			LLIN only		Children (6413)	Provider	Economic	4.86	per person covered (assumes 2 ITN per child)
Makoutode et al (2014)[113]	Benin	Urban, rural	LLIN	Campaign	Households (6951-13017)	Societal	Economic	6.53-6.58	per net distributed across settings
		Urban, rural	IRS	Campaign		Societal	Economic	4.69	per person protected across all settings
		Urban	LLIN, IRS	Campaign		Societal	Economic	91.24-200.54	per case averted compared to LLIN only across settings

		Rural	LLIN IRS	Campaign		Societal	Economic	37.36-409.49	per case averted compared to LLIN only across settings
Rezaei-Hemami et al (2014)[114]	Iran		Larviciding	Various	500 people	Provider	NR	2.58	per person covered
			IRS		500 people			1.21	per person covered
			LLIN		500 people			1.80	per person covered
			Surveillance		500 people			6.53	per person covered
			Border facilities		500 people			10.39	per person covered
Smith Gueye et al (2014)[115]	Namibia	Mixed	Diagnosis, treatment, LLIN, IRS, surveillance, monitoring of cross-border activities	Various	3 districts (NR)	Provider	NR	3.88- 8.47	per person covered across endemicity areas
Stelmach et al (2018)[116]	Tanzania (mainland)	Mixed	ITN, IRS	Various	Nationwide (NR)	Societal	Economic	8.39	per person
			ITN only			Societal	Economic	3.82	per person
			ITN, IRS			Societal	Economic	170.64	per additional case averted with ITN and IRS compared to ITN only
Sun et al (2015)[117]	China	Mixed	Passive and active case detection, MDA, ITN, IRS	Public health facilities		Provider	Financial	140.97	per case averted compared to no elimination programme

Note: *includes IRS, ITN, LSM, diagnostics, treatment; ITN: insecticidal-treated nets; LLIN: long-lasting insecticidal treated nets; IRS: Indoor residual spraying; LSM: larval source management; CE: cost-effectiveness; DALY: disability adjusted life year
